# Gut Microbial Genetic Variation Prolongs Host Healthy Longevity and Remodels Metabolome and Proteome in *Drosophila Melanogaster*

**DOI:** 10.1002/advs.202505469

**Published:** 2025-09-26

**Authors:** Liying Wang, Ziling Yang, Yulu Pang, Xinyue Wu, Xinrong Zhang, Tao Zhu, Xiaoyue Ding, Wei Liu, Yuan Zhou, Pengcheng Zhang, Ying Li, Zuobin Zhu

**Affiliations:** ^1^ Jiangsu Engineering Research Center for Precision Diagnosis and Treatment of Polygenic Critical Diseases Key Laboratory of Genetic Foundation and Clinical Application Department of Genetics Xuzhou Medical University Xuzhou 221000 China; ^2^ Jiangsu Key Laboratory of Brain Disease and Bioinformation Research Center for Biochemistry and Molecular Biology Xuzhou Medical University Xuzhou 221004 China; ^3^ Medical Technology College Xuzhou Medical University Xuzhou 221000 China

**Keywords:** anti‐aging, genetic variation, gut microbiota, longevity, metabolic reprogramming

## Abstract

Microbial genetic variation plays a crucial role in shaping host‐microbe interactions; however, its impact on healthy aging remains largely unexplored. This study investigates how genetic variations in gut‐residing *Saccharomyces cerevisiae* affect the health and lifespan of *Drosophila melanogaster*. This study identifies 14 *yeast* mutants that significantly extended the lifespan of *D. melanogaster*, with 13 mutants enhancing locomotor function in aged flies and two mutants improving reproductive capacity. Metabolomic and proteomic analyses reveal that these mutant yeasts rejuvenate the metabolic state of the aging gut and alter protein levels in tissues outside the gut. Most of the proteins with at least a two‐fold change are upregulated. The data also highlights mitochondrial energy metabolism as a key anti‐aging mechanism driven by the yeast. Notably, terpenoid metabolites such as ergosterol acetate showed strong lifespan‐extending effects and may influence energy metabolism. In conclusion, these findings establish a strong link between gut metabolic status and healthy aging, underscoring the significance of the microbial‐host mitochondrial axis as a key mechanism by which gut microbes promote host health and longevity. Furthermore, genetically engineered probiotics in model organisms offer a promising potential strategy for extending healthy lifespan, thus meriting further investigation in translational research models.

## Graphical abstract

This study reveals that specific genetic variations in gut‐residing Saccharomyces cerevisiae significantly extend healthspan and lifespan in Drosophila melanogaster. These mutants rejuvenate aged intestinal metabolism, upregulate mitochondrial proteins, and enhance energy pathways. The terpenoid metabolite ergosterol acetate is identified as a key longevity‐promoting compound. These findings highlight the microbial‐mitochondrial axis as a central mechanism in healthy aging.

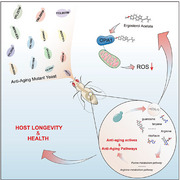

## 1 Introduction

The gut microbiota plays a pivotal role in human health and aging, with significant implications for anti‐aging research.^[^ ^1^ ^]^ Exploring the potential of gut microbes in this field holds considerable promise.^[^ ^2^, ^3^ ^]^ Previous studies have demonstrated that fecal microbiota transplantation (FMT) can improve physiological functions and extend lifespan in mice, suggesting a direct influence of the gut microbiome on aging.^[^ ^4^, ^5^ ^]^ However, understanding the precise molecular mechanisms by which gut microbes regulate host aging remains challenging due to the complexity of the microbial community. Although FMT has shown therapeutic potential for various diseases, its application is hindered by challenges such as standardization and quantification of its effects.^[^ ^6^, ^7^ ^]^ Although efforts to enhance gut microbiota composition using probiotic formulations are ongoing, their efficacy remains limited. The insufficient characterization of probiotic strains and a lack of clarity regarding the mechanisms linking gut microbiota to human health continue to obstruct progress in evaluating their therapeutic benefits,^[^ ^8^ ^]^ emphasizing the need for innovative probiotic strategies.

Recent findings suggest that the host's genetic background significantly influences gut microbial diversity, positioning the microbiota as a “second genome” and a dynamic gene pool.^[^ ^9^, ^10^ ^]^ Despite this, the extent to which microbial genetic variation impacts host health remains poorly understood. Earlier research on gut microbe genetics primarily centered on microbial toxicity and viability. However, emerging evidence points to genetic variations in gut microbes as factors influencing host lifespan, growth, and development,^[^ ^11^, ^12^, ^13^, ^14^ ^]^ Thus, targeting specific genes within the broader microbial population may offer a more precise and effective means of modulating gut microbiota function, surpassing the current approaches of probiotics and FMT.

Throughout coevolution, the principle of survival of the fittest has driven mutual adaptation between gut microbiota and their hosts.^[^ ^15^, ^16^ ^]^ As a result, understanding the evolutionary dynamics of host‐microbiota relationships has become a key focus for gut microbiota research in the coming decade.^[^ ^17^ ^]^ This raises the question of whether genetic variations within gut microbiota significantly influence host survival and play a role in driving host evolution.^[^ ^18^ ^]^ However, studying these variations in their natural context is challenging due to the complexity of the gut microbiota and the time required for mutations to develop. *Saccharomyces cerevisiae* and its metabolites are known to have a significant impact on animal and human health.^[^ ^19^, ^20^ ^]^ Recent studies have demonstrated that genetically engineered *S. cerevisiae* can suppress intestinal inflammation, reduce fibrosis, and restore microbial balance.^[^ ^21^ ^]^ Thus, genetic variations in specific *S. cerevisiae* genes may produce metabolites that confer greater health benefits to the host. In anti‐aging research, it is crucial to screen genetically modified *S. cerevisiae* strains capable of extending host longevity and to investigate their regulatory mechanisms.

The gut microbiota of *Drosophila melanogaster* is relatively simple, making it an ideal model for studying the interactions between gut microbes and host lifespan.^[^ ^22^, ^23^ ^]^ Our previous research revealed that genetic variations in *S. cerevisiae*, a *yeast* commonly used in winemaking, could influence sleep patterns and markedly reduce the lifespan of *fruit flies*.^[^ ^11^ ^]^ *S. cerevisiae* contains numerous beneficial active compounds.^[^ ^24^ ^]^ In this study, *D. melanogaster* was fed with various *S. cerevisiae* single‐gene knockout strains to identify those capable of extending the lifespan of male and female flies without impairing their movement or reproductive abilities. This approach offers potential for developing genetically modified microbial interventions in model systems and represents a research avenue for modulating aging‐associated pathways. Through non‐targeted metabolomics, this study analyzed the metabolic changes in both cultured anti‐aging *S. cerevisiae* and the gut at different life stages after feeding mutant strains, along with proteomic changes in long‐lived flies. This allowed us to identify active substances contributing to anti‐aging effects and the metabolic signatures associated with extended longevity.

## 2 Results

### 2.1 A High‐Throughput Screen for *S. cerevisiae* Single Gene Deletions That Prolong the Health Lifespan of *D. melanogaster*

**Figure 1:**
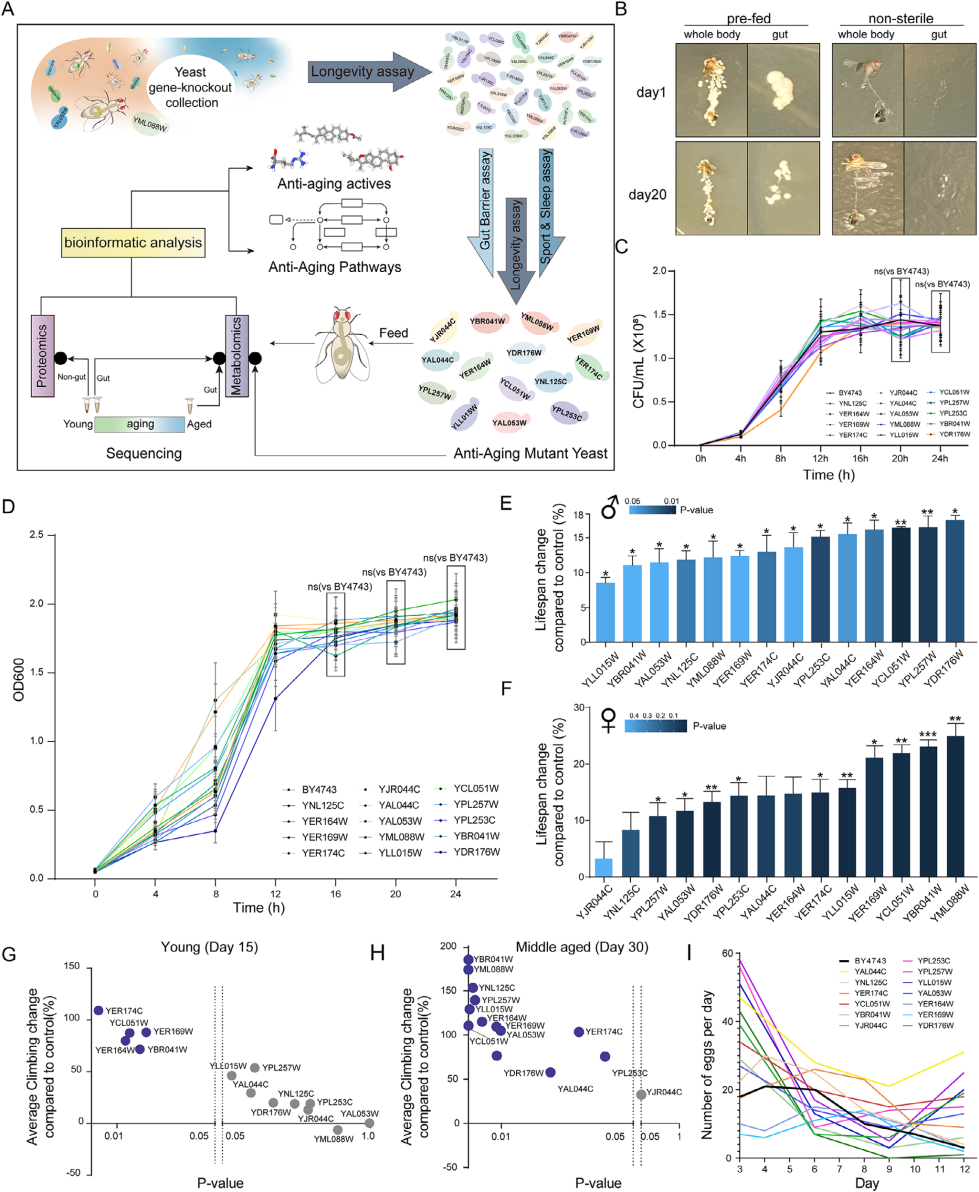
Impact of Anti‐Aging Gut Microbiota Strains on Lifespan and Behavior. A) Workflow depicting the construction and screening of mutant *Saccharomyces cerevisiae* strains using a *Drosophila melanogaster* platform. The diagram highlights key steps, from *yeast* mutation to the completion of the process. **B)** Growth of *S. cerevisiae* colonies (*BY4743* strain as an example) in the *Drosophila* intestinal tract after 24 h of exposure to YPD medium. **C)** Growth curves showing colony‐forming unit (CFU) changes of 14 mutant yeast strains and the control strain BY4743 at different time points. Data are presented as mean ± SD (*n* = 6). Statistical significance was determined by Student's t‐test; ns indicates no significant difference. **D)** Growth curves showing optical density (OD) changes of 14 mutant yeast strains and the control strain *BY4743* at different time points. Data are presented as mean ± SD (*n* = 6). Statistical significance was determined by Student's t‐test; ns indicates no significant difference. **E)** Bar chart showing the percentage change in the lifespan of male *Drosophila* fed with each of the 14 mutant strains, compared to the wild‐type control, validated through triplicate experiments (*n* = 80 per experiment). Asterisks represent statistical significance (^*^ *p* < 0.05, ^**^ *p* < 0.01, and ^***^ *p* < 0.001) based on t‐tests. **F)** Bar chart illustrating the impact of 14 mutant strains on female *Drosophila* lifespan (n = 80 per experiment). Asterisks represent statistical significance (^*^ *p *< 0.05, ^**^ *p* < 0.01, and ^***^ *p* < 0.001) based on t‐tests. **G,H)** Scatter plots of changes in climbing ability in *Drosophila* fed with mutant *yeast* strains during youth **G**) and middle age **H**), compared to the wild‐type control. Blue dots denote significant changes; gray dots indicate no significant difference (4 group, 3 replicates.). **I)** Line chart showing daily egg‐laying trends of *Drosophila* fed with different *yeast* strains. Asterisks represent statistical significance (^*^ *p* < 0.05, ^**^ *p* < 0.01, and ^***^ *p* < 0.001) based on *t*‐tests (10 flies per group, with 3 replicates).

Nearly 2,000 single‐gene homozygous knockout strains of *S. cerevisiae* were administered to *D. melanogaster* to assess their effects on lifespan, intestinal barrier function, and locomotion. Subsequent analysis of metabolic and protein expression profiles in the intestinal tissues of *D. melanogaster* exposed to life‐extending mutant *yeast* enabled the identification of metabolic and proteomic signatures linked to longevity, as well as the discovery of bioactive compounds with anti‐aging properties (**Figure** **1**A).

The initial objective was to assess whether *S. cerevisiae* could establish persistent colonization in the gut of *D. melanogaster*. Adult flies were pre‐fed for three days before transitioning to a *yeast*‐free environment. The *yeast* demonstrated stability in the gut for over two weeks (Figure 1B). Colony‐forming unit (CFU) counts demonstrated no significant difference between wild‐type and mutant strains during the stationary phase, indicating comparable viable cell numbers (Figure 1C; Figure S1A, Supporting Information). Similarly, growth curves based on OD_600_ measurements at various time points showed no significant difference in the stationary phase, suggesting both strains reached comparable cell densities (Figure 1D; Figure S1B, Supporting Information). The CFU counts of yeast in the gut of *Drosophila* showed no significant difference between the young and elderly groups after yeast feeding (Figure S1E,F, Supporting Information). After screening, 14 *S. cerevisiae* mutants were found to significantly extend the lifespan of male *D. melanogaster*, with 13 mutants showing lifespan increases exceeding 10% (Figure 1E; Table S1 and Figure S1G, Supporting Information). Further investigation into their impact on female flies revealed that 10 of these mutants markedly extended female lifespan, while the remaining 4 showed moderate, statistically insignificant increases (Figure 1F). This suggests a sexual dimorphism in the influence of *S. cerevisiae* mutants on lifespan extension in *D. melanogaster*.

Extending lifespan without improving the quality of life for the elderly would only increase societal burdens. Previous studies have shown that lifespan extension alone does not necessarily prevent age‐related behavioral decline.^[^ ^25^, ^26^, ^27^, ^28^ ^]^ Thus, improving health during aging is more critical than merely extending lifespan. The 14 longevity‐promoting *S. cerevisiae* mutants were further evaluated for their effects on motor function. Compared to wild‐type controls, *YER174C, YER164W, YCL051W, YBR041W*, and *YER169W* significantly enhanced the climbing ability of young flies (Figure 1G,;Table S1, Supporting Information). More notably, all 14 mutants improved the climbing ability of middle‐aged flies (Figure 1H; Table S1, Supporting Information).

Research has often highlighted a trade‐off between reproduction and longevity, typically showing a negative correlation between the two.^[^ ^28^, ^29^, ^30^ ^]^ However, *YAL044C*, *YCL051W*, and other *S. cerevisiae* mutants not only preserved reproductive abilities in *D. melanogaster* but also extended their reproductive cycle (Figure 1I). These results suggest that achieving both longevity and reproductive success is possible through effective interventions.

### 2.2 The *S. cerevisiae* Mutants Enhance the Intestinal Barrier Function in Aged *D. melanogaster*

**Figure 2:**
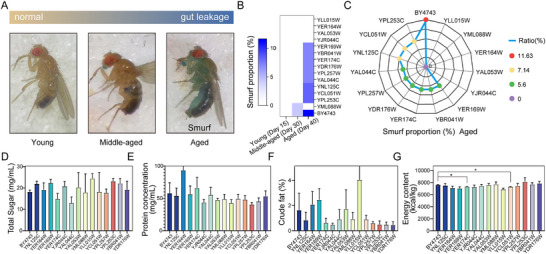
Effects of Mutated *Yeast* Strains on the Intestinal Barrier of *Drosophila*. A) Schematic showing gut leakage (Smurf phenotype) as *Drosophila* age. **B)** Proportion of *Drosophila* displaying the Smurf phenotype at various life stages after feeding with anti‐aging mutant yeast strains. The proportion is shown for each life stage, indicating the phenotype prevalence at different time points (*n* = 40 per group). **C)** Proportion of Smurf phenotype Drosophila at advanced age after feeding with mutant yeast strains (*n* = 40). This bar chart represents the cumulative proportion of individuals exhibiting the Smurf phenotype by the end of the study period. D–G) Total sugar D), protein concentration E), energy content G), and crude fat F) in 15 yeast strains. Total sugar: three biological replicates, each with three technical replicates (mean ± SEM). Protein concentration: four biological replicates, each with three technical replicates (mean ± SEM). Energy content and crude fat: three biological replicates (mean ± SD). Statistical significance was assessed by Student's *t*‐test, ^*^ *p* < 0.05.

The intestinal barrier function is a key indicator of gut health.^[^ ^31^ ^]^ As *D. melanogaster* age, their gut barrier function deteriorates, leading to intestinal leakage (**Figure** **2**A). A pertinent question is whether the anti‐aging *S. cerevisiae* mutants can mitigate intestinal leakage in elderly *D. melanogaster*. Using the Smurf Phenotype Assay, the effect of 14 anti‐aging *S. cerevisiae* strains on the integrity of the intestinal barrier was examined. The results indicated that, while the mutants did not compromise intestinal barrier function in young flies, they showed a notable ability to alleviate age‐related intestinal leakage in older flies (Figure 2B,C). To determine whether nutritional differences contributed to lifespan extension, total sugar, protein concentration, and crude fat were measured in mutant strains compared to the control (Figure 2D–F; Table S1, Supporting Information). No significant differences were observed in these parameters. Energy content was reduced in *YER174C* and *YCL051W* mutants (Figure 2G; Table S1, Supporting Information).

However, flies fed with heat‐killed mutant strains did not exhibit any lifespan extension compared with those fed wild‐type strains (Figure S1C,D, Supporting Information). These findings suggest that alterations in nutritional composition alone in *YER174C* and *YCL051W* mutants are insufficient to account for the lifespan‐extending phenotype of *D. melanogaster*.

### 2.3 Yeast Mutants Reshape the *D. melanogaster* Metabolome

**Figure 3:**
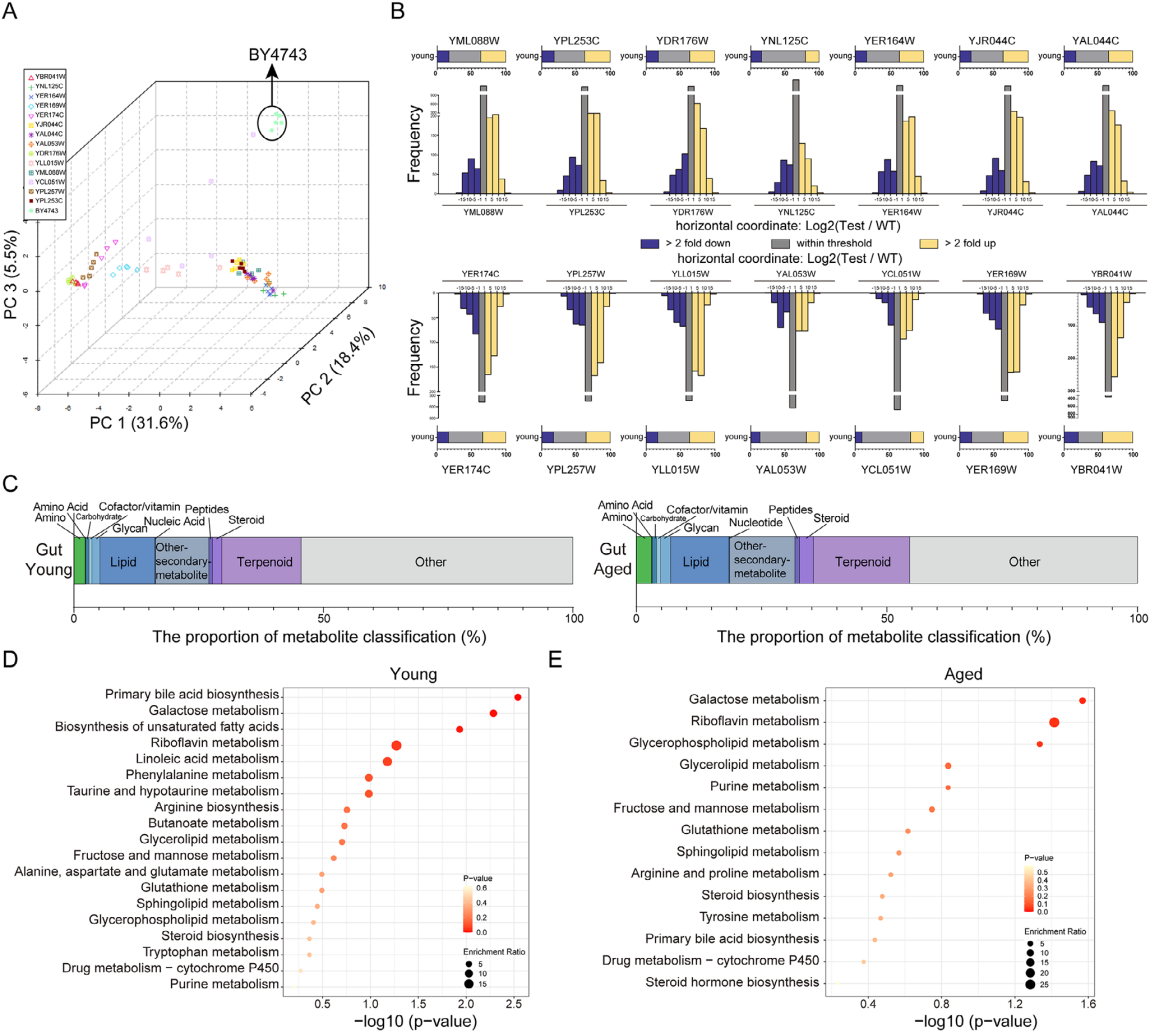
*Yeast* Mutants Reshape the *Drosophila* Metabolome. A) PCA plot illustrating gut metabolome profiles of young *Drosophila* fed with anti‐aging mutant *yeast* strains. Each point represents an individual sample, with separation between metabolic profiles. The reference strain *BY4743* is highlighted. **B)** Bar chart showing significant changes in gut metabolites of young *Drosophila* fed with mutant *yeast* strains compared to the control *BY4743* strain. Yellow bars indicate upregulated metabolites, while blue bars represent downregulated ones. A vertical bar chart shows the distribution of metabolites across varying significance levels. **C)** Pie chart classifying significantly altered gut metabolites in *Drosophila* fed with anti‐aging mutant *yeast* strains. **D)** Bubble diagram illustrating KEGG pathway enrichment for differential metabolites. The x‐axis shows the ‐log10(*p*‐value) of each pathway, and the size of the bubbles represents the relative abundance of metabolites associated with each pathway.

The gut serves as the primary site of microbial activity in the host, making it critical to understand how anti‐aging mutant *S. cerevisiae* modulates the metabolic state of the gut in *D. melanogaster* for investigating health and longevity. A total of 955 metabolites were identified using liquid mass spectrometry in conjunction with the MassBank database. Principal component analysis (PCA) revealed clear differentiation between the intestinal metabolites of young flies fed control *yeast BY4743* and those fed the 14 anti‐aging mutant strains (**Figure** **3**A), suggesting that alterations in gut metabolism are a key mechanism through which these mutant *S. cerevisiae* exert their anti‐aging effects. Further analysis showed significant increases or decreases in metabolite abundance in 887 metabolites during the young stage and 790 during the old stage in flies fed the mutant *S. cerevisiae* (*p* < 0.05, |LogFC| > 1) (Figure S2A and Table S2, Supporting Information). Notably, the number of up‐regulated metabolites in the gut microbiota of young flies fed the mutant strains was more than double that of down‐regulated metabolites (Figure 3B).

To explore the gut metabolic changes in long‐lived flies, metabolites that displayed more than a two‐fold change in abundance across at least three long‐lived flies were functionally classified. During youth, 645 metabolites were predominantly enriched in terpenoids (13.49%), other secondary metabolite lipids (10.85%), lipids (12.25%), amino acids (1.86%), and other compounds (56.12%) (Figure 3C; Table S3, Supporting Information). In aged flies, 431 metabolites were primarily enriched in terpenoids (17.40%), other secondary metabolite lipids (11.60%), lipids (12.30%), amino acids (3.48%), and other compounds (47.56%) (Figure 3C; Table S3, Supporting Information). KEGG pathway analysis of youth‐specific metabolites revealed significant enrichment in pathways related to primary bile acid biosynthesis, galactose metabolism, and riboflavin metabolism (Figure 3D,E). In aged flies, functional enrichment and KEGG pathway analysis showed a predominant involvement of glycerolipid metabolism and galactose metabolism (Figure 3D,E).

### 2.4 Yeast Mutants Enhance the Intestinal Metabolic Status of Aged *D. melanogaster*

**Figure 4:**
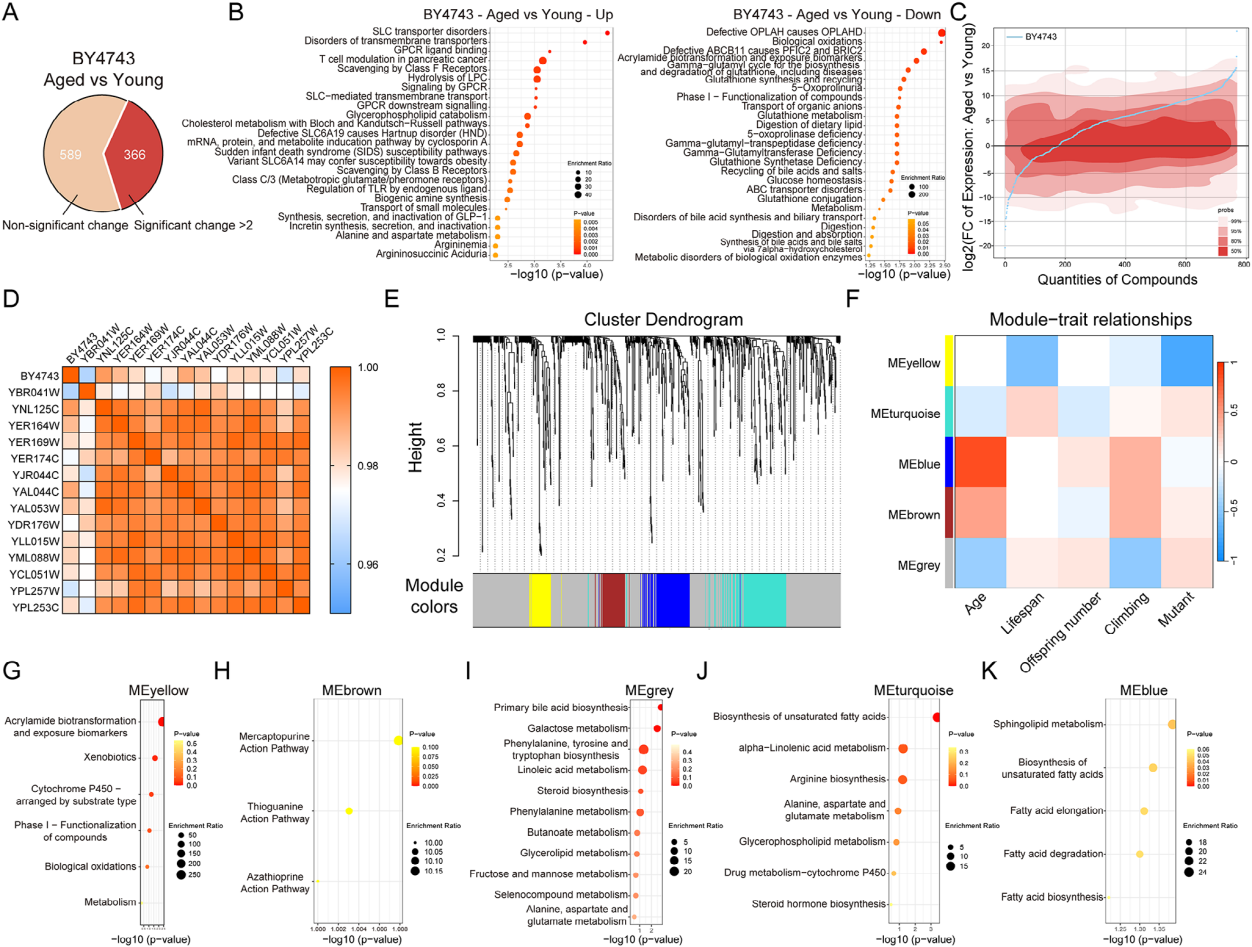
Alteration of Intestinal Metabolic Status in Aged *Drosophila* by *Yeast* Mutants. A) Venn diagram showing the number of significantly different metabolites between young and aged *Drosophila* fed with the control *yeast* strain. **B)** Bubble plot depicting KEGG enrichment analysis for significantly altered metabolites between young and aged *Drosophila* fed with the control *yeast* strain. **C)** Ratio changes of gut metabolites in aged *Drosophila* fed with anti‐aging mutant *yeast* strains compared to young flies fed the control strain. The *y*‐axis represents the log2 fold change (LogFC) of metabolite levels in aged flies fed with mutant strains versus young flies fed with control *yeast* (*BY4743*). The blue line represents the control group, while the red shading indicates mutant groups with varying confidence intervals. **D)** Heatmap showing the correlation of metabolite ratios between the 14 mutant strains and the control group. The data represent metabolite ratios from aged *flies* compared to young control *flies*. **E)** Clustering dendrogram of genes, with dissimilarity measured by topological overlap. The dendrogram shows gene clustering based on the similarity of their expression profiles, with each branch representing a gene module. The module colors are assigned based on the KEGG pathway enrichment, indicating the biological pathways that the genes are associated with. **F)** Module‐trait associations, where each row represents a gene module and each column corresponds to a trait. The table is color‐coded by correlation, according to the color legend. **G–K)** Bubble plot illustrating metabolite enrichment results within the five gene modules.

Extensive research has repeatedly demonstrated that aging significantly reshapes metabolic processes.^[^ ^32^, ^33^, ^34^ ^]^ Over one‐third of the quantified metabolites exhibited at least a two‐fold alteration in aged *D. melanogaster* when compared to younger specimens (**Figure** **4**A). Specifically, 298 metabolites showed a two‐fold increase, primarily linked to SLC transporter disorders, transmembrane transporter dysfunction, and GPCR ligand binding pathways. In contrast, 68 metabolites experienced a two‐fold decrease, mainly enriched in pathways such as Defective OPLAH causing OPLAHD and Biological oxidations (Figure 4B). To assess whether the gut metabolic state in advanced age could alleviate these age‐related metabolic shifts, this study compared the intestinal metabolic profile of control flies at a young age as a reference. Flies fed with anti‐aging mutant *S. cerevisiae* exhibited an intestinal metabolic state more closely resembling the youthful baseline compared to older flies fed with control *S. cerevisiae* (Figure 4C). Additionally, the intestinal metabolic states of the 14 long‐lived flies fed mutant *S. cerevisiae* showed greater homogeneity when compared to aged control flies (Figure 4D). Further analysis revealed that of the 298 metabolites up‐regulated during aging, more than 120 were reversed in at least three long‐lived flies (Table S3 and Figure S2B, Supporting Information). Similarly, 17 out of the 68 down‐regulated metabolites during aging were reversed in more than three long‐lived flies (Table S3 and Figure S2B, Supporting Information). These findings suggest that anti‐aging mutant *S. cerevisiae* significantly restored the metabolic state of older flies, aligning it more closely with the youthful intestinal metabolic profile.

Among the 14 identified anti‐aging mutant *yeast*s, most enhanced *Drosophila* motility without impairing reproductive capacity. This raises the question: do these age‐related phenotypes consistently reflect signatures in intestinal metabolites?

Weighted gene co‐expression network analysis (WGCNA) identified five distinct phenotypic modules associated with aging (Figure 4E,F; Table S3, Supporting Information). Compared to traditional differential analysis, WGCNA offers a more comprehensive and systematic approach to uncovering the underlying patterns and biological significance in expression data. It is particularly suitable for exploring interactions within complex biological systems. In this study, the omics data followed the distribution pattern typical of high‐throughput datasets and included phenotypic modules that could be correlated. Therefore, WGCNA was applied to cluster genes with different expression patterns into several distinct color‐coded modules (Figure 4E). This analysis revealed the correlation between aging and the different modules (Figure 4F). The metabolic enrichment analysis for each module highlighted key pathways influencing phenotypes; for instance, fatty acids strongly impacted motility, while linolenic acid and arginine were positive regulators of lifespan. Moreover, primary bile acids and galactose played essential roles in reproduction (Figure 4G–K).

### 2.5 Yeast Mutants Extensively Reshape the *D. melanogaster* Proteome

**Figure 5:**
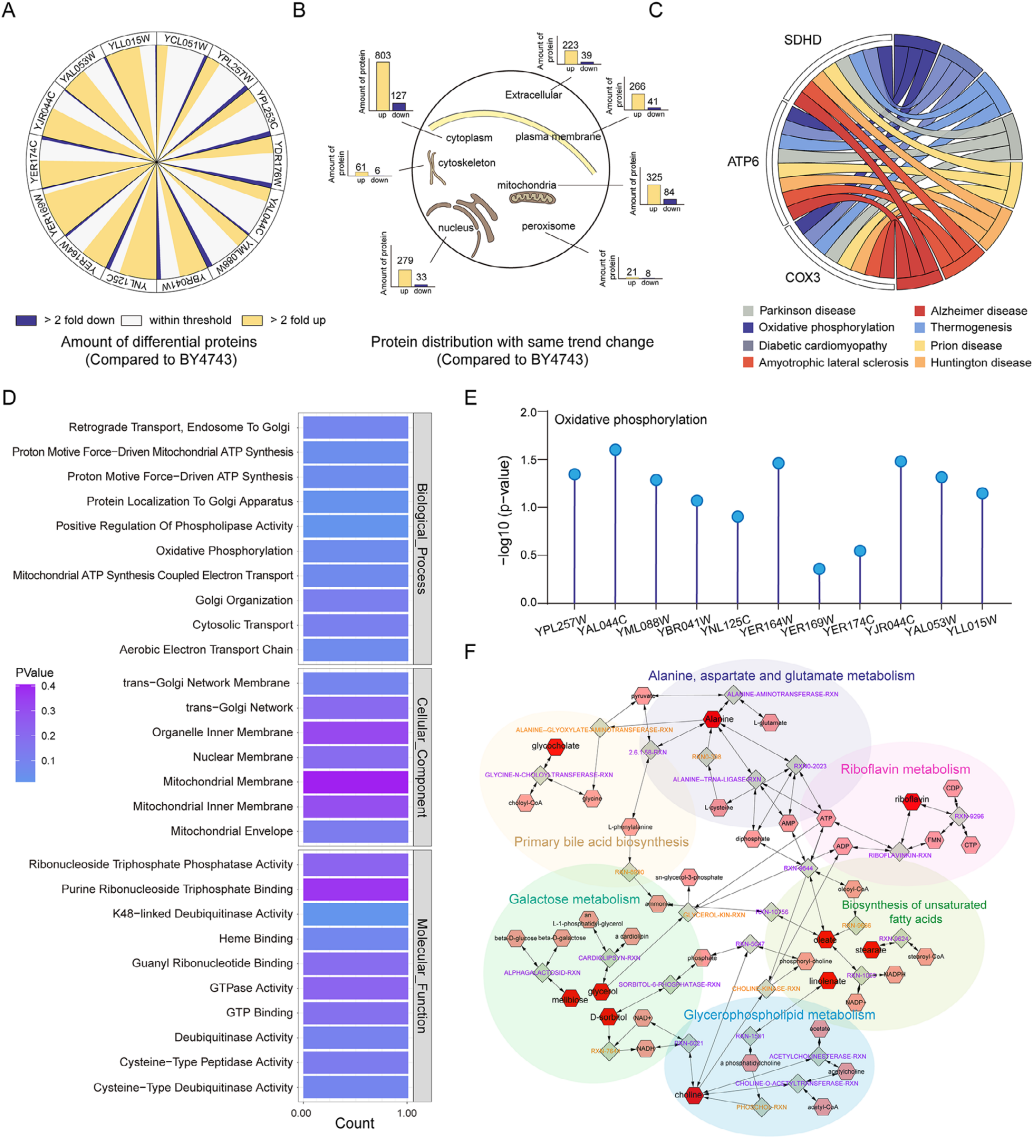
Proteome Changes in *Drosophila* Extra‐Intestinal Tissues. A) Proportional chart showing the proteins significantly affected in non‐gut tissues of *Drosophila* by different mutant *yeast* strains. The outer ring labels each mutant strain group. Yellow segments represent the number of proteins significantly upregulated in long‐lived flies compared to the control group, while blue segments indicate downregulated proteins (*p* < 0.05). Differential analysis was performed using a two‐tailed *t*‐test, with each experimental group compared to the control group. The sizes of the segments correspond to the proportion of affected proteins. **B)** Chart depicting proteome changes in subcellular compartments of long‐lived *Drosophila*. Yellow represents proteins upregulated by more than two‐fold, and blue represents proteins downregulated by less than 0.5‐fold across various subcellular compartments. **C,D)** Enrichment results of proteins upregulated (greater than two‐fold) in over half of the long‐lived *Drosophila*. **C)** Chord diagram showing the connections between KEGG pathways and enriched metabolites linked to these pathways. **D)** Bar chart illustrating GO functional enrichment results, with p‐values indicated in the legend. **E)** Enrichment of mitochondrial oxidative phosphorylation in the functional analysis of differential proteins between long‐lived *Drosophila* and the control group. The figure highlights the *p*‐values for oxidative phosphorylation pathway enrichment across different groups. **F)** Graphical representation of the interactions between differential metabolites and associated pathways using FlyScape. Red hexagons represent differentially expressed metabolites of interest, pink hexagons represent metabolites not measured or not relevant to the pathway, and gray diamonds denote reactions.

This study identified 3063 proteins in wild‐type fly tissues and 3390 proteins in the tissues of flies fed with mutant *S. cerevisiae*. Compared to the control strain *BY4743*, a substantial portion of proteins in the parental tissues of 14 flies fed with anti‐aging mutant *S. cerevisiae* exhibited more than a two‐fold change in expression (Table S2, Supporting Information). Remarkably, about half of the proteins in eight mutant *S. cerevisiae*‐fed flies demonstrated a two‐fold upregulation or downregulation relative to the control group. Notably, the majority of upregulated proteins were found among the differential proteins in flies fed with 14 anti‐aging mutant *yeast*s (**Figure** **5**A).

Proteomic analysis revealed significant alterations in the composition and relative abundance of proteins across various cellular compartments, including the cytoplasm, extracellular space, mitochondria, and nucleus (Figure 5B). A similar pattern of proteomic shifts was also observed in long‐lived nematodes.^[^ ^35^ ^]^ Further proteomic profiling of 14 anti‐aging mutant *S. cerevisiae* and control *S. cerevisiae*‐fed flies led to the identification of 19 proteins that were upregulated by at least two‐fold in over half (≥ 50%) of the long‐lived flies. KEGG pathway and GO function enrichment analyses indicated that these 19 proteins were significantly enriched in mitochondrial oxidative phosphorylation and related pathways (Figure 5C,D; Table S4, Supporting Information). Moreover, when comparing differential protein functions across long‐lived *Drosophila* species and control groups, mitochondrial oxidative phosphorylation was enriched in over 80% of the groups (11 out of 14) (Figure 5E; Table S4, Supporting Information). Consistently, several key KEGG pathways enriched by intestinal metabolites in long‐lived *D. melanogaster* were closely linked to mitochondrial energy metabolism pathways (Figure 5F). These results underscore the pivotal role of mitochondrial pathways as a core anti‐aging mechanism mediated by intestinal microbes.

### 2.6 The Global Metabolome View of the Mutation Yeast

**Figure 6:**
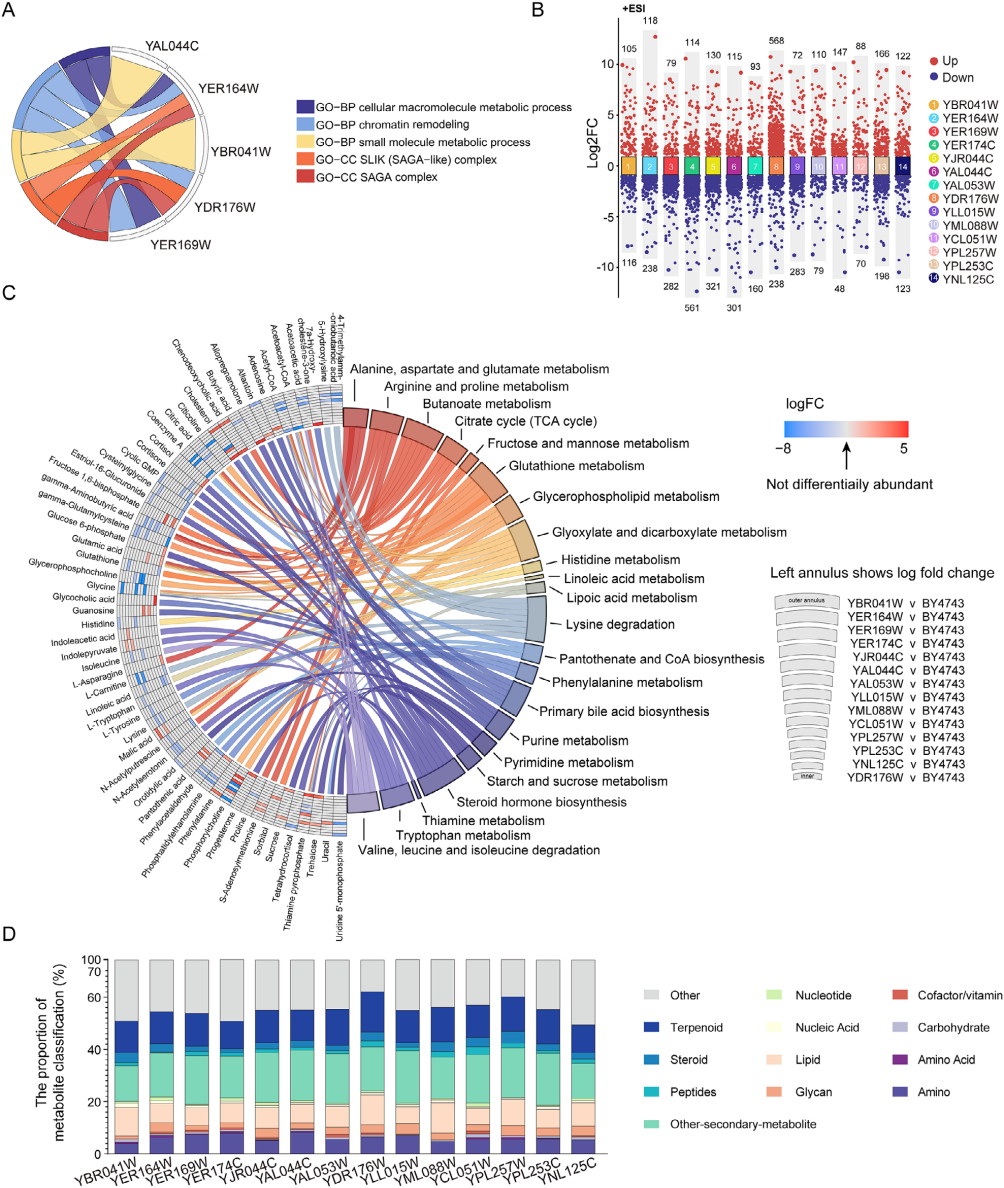
Metabolic Recombination in Mutant Strain Metabolomes. A) Chord diagram illustrating Gene Ontology (GO) enrichment results for the mutated genes in the 14 anti‐aging mutant strains. The diagram shows connections between GO terms and corresponding genes. **B)** Volcano scatter plot of metabolite levels in mutant strains compared to control in positive ion modes. Each point represents the difference and significance of metabolite levels, with red indicating significantly upregulated metabolites and blue indicating significantly downregulated metabolites. **C)** Multi‐group chord diagram depicting significant pathway enrichment and corresponding metabolites across mutant strains. Each section corresponds to a mutant strain, linking metabolites and pathways from the outer to the inner circle. **D)** Stacked bar chart showing the distribution of metabolite classes within significantly altered metabolites (adjusted *p*‐value < 0.05, fold change ≥ 2) for each mutant strain relative to the wild‐type *BY4743* strain. Each bar represents a mutant strain, with segments depicting the proportional composition of various metabolite classes among the significantly altered metabolites.

The 14 genes from *S. cerevisiae* underwent GO gene functional enrichment analysis, which revealed significant enrichment in cellular macromolecule metabolic processes, chromatin remodeling, small molecule metabolism, and other biological processes. These results suggest that these mutant *S. cerevisiae* may influence host lifespan by modulating metabolite abundance (**Figure** **6**A). Additionally, metabolomic profiling of the control *S. cerevisiae* and the 14 mutant strains demonstrated varying levels of metabolic changes across the mutants compared to the control strain *BY4743* (Figure 6B; Table S2, Supporting Information) (*p* < 0.05, |LogFC| >1). Pathway enrichment and statistical analysis highlighted consistent disruptions in energy metabolism‐related pathways across several mutants (Figure 6C; Table S5, Supporting Information), with Lipid, Terpenoid, and Other‐secondary‐metabolite categories prominently emerging as differential metabolites (Figure 6D). Notably, terpenoids stood out as promising candidates for drug development due to their well‐known anti‐cancer and anti‐aging properties.

### 2.7 Elevated Ergosterol Acetate Expression Mitigates Aging by Enhancing Mitochondrial Function And Reducing Reactive Oxygen Species Levels

**Figure 7:**
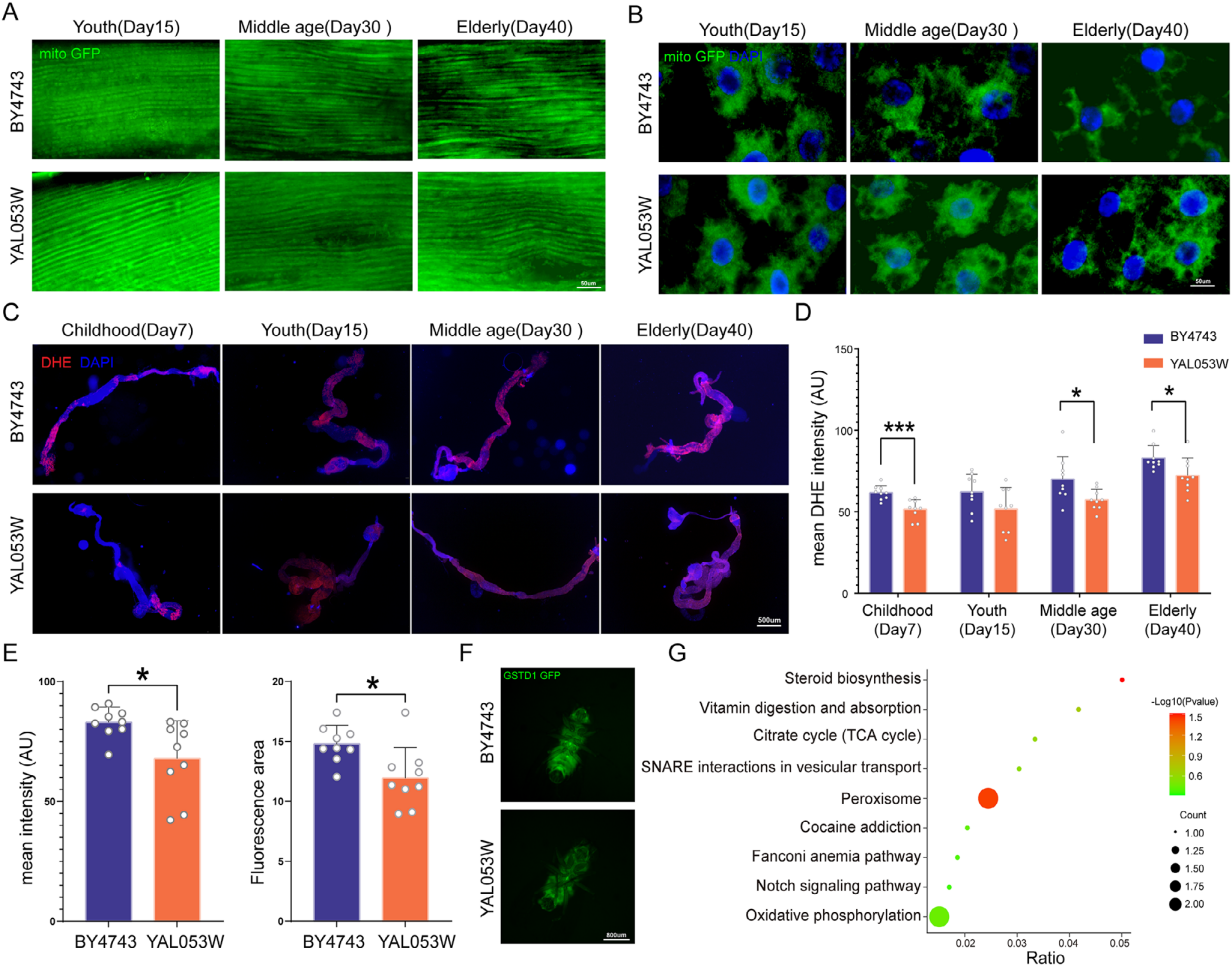
Investigating the Regulation of Host Mitochondrial Status and Oxidative Stress by Mutant Strains. A) Mitochondrial distribution in the flight muscles of *Drosophila* fed *BY4743* and *YAL053W* strains at different ages. GFP‐labeled mitochondria are shown at 40X magnification (*n* = 3). **B)** Mitochondrial aggregation in the fat bodies of *Drosophila* fed *BY4743* and *YAL053W* at different ages, with GFP‐labeled mitochondria (green) and DAPI (blue) shown at 40X magnification (*n* = 3). **C)** ROS levels in the gut of *Drosophila* at different ages fed with wild‐type control (*BY4743*) and *YAL053W* strains. Red indicates higher ROS levels, while DAPI is shown in blue (4X magnification)(*n* = 9). **D)** Statistical analysis of red fluorescence intensity in the gut, measured using ImageJ. *n* = 9; *p* < 0.05 based on Student's *t*‐test. **E)** Statistical analysis of fluorescence intensity and range in *Drosophila*. Average fluorescence intensity and range were calculated using ImageJ. *n* = 9; *p *< 0.05 based on Student's *t*‐test. **F)** Visualization of ROS levels in *Drosophila* using fluorescence microscopy. GFP‐labeled GSTD1 indicates ROS levels, with brighter green fluorescence indicating higher ROS levels (2.5 × magnification)(*n* = 9). **G)** Bubble plot showing enrichment of proteins significantly upregulated in *Drosophila* fed with the *YAL053W* strain compared to the wild‐type control.

A previous study demonstrated that mutant *yeast* induces aging in *fruit flies* by increasing oxidative stress in the gut.^[^ ^11^ ^]^ In the current study, a significant upregulation of mitochondrial function‐related proteins was observed in flies fed with mutant *S. cerevisiae*. To assess whether anti‐aging mutant *S. cerevisiae* affects mitochondrial quantity and distribution in striated muscle and adipose tissue, UAS‐mito‐GFP was used to visualize mitochondria in *D. melanogaster*.^[^ ^36^ ^]^ Mitochondrial changes in these tissues directly reflect fly mobility and physiological health, indirectly indicating shifts in energy metabolism and overall well‐being. Our findings show that as flies age, mitochondria in striated muscle become increasingly irregular and fragmented (Figure 7A), while both the number and aggregation of mitochondria in adipose tissue markedly decline (Figure 7B). Remarkably, YAL053W was able to mitigate these mitochondrial abnormalities, improving distribution in both muscle and adipose tissue and delaying age‐related mitochondrial decline in adipose tissue (**Figure** **7**A,B).

There is a well‐established reciprocal relationship between mitochondrial damage during aging and excessive ROS production.^[^ ^10^ ^]^ Previous studies have linked mitochondrial ROS with metabolic pathways.^[^ ^37^ ^]^ In our experiment, a significant reduction in ROS levels was observed in the intestinal tissues of flies fed with the mutant strain *YAL053W* (Figure 7C,D). Moreover, in vivo imaging of GSTD1‐GFP‐marked flies, where stronger green fluorescence indicates higher reactive oxygen species (ROS) levels, revealed a pronounced decrease in ROS levels in flies fed this strain (Figure 7E,F). Proteomic analysis of flies fed *YAL053W* indicated a significant enrichment in the peroxisome pathway, with key proteins such as Gnpat and Pex14 significantly upregulated in the experimental group (Figure 7G).

### 2.8 Yeast Ergosterol Acetate Overproduction as a Longevity‐Promoting Mechanism

**Figure 8:**
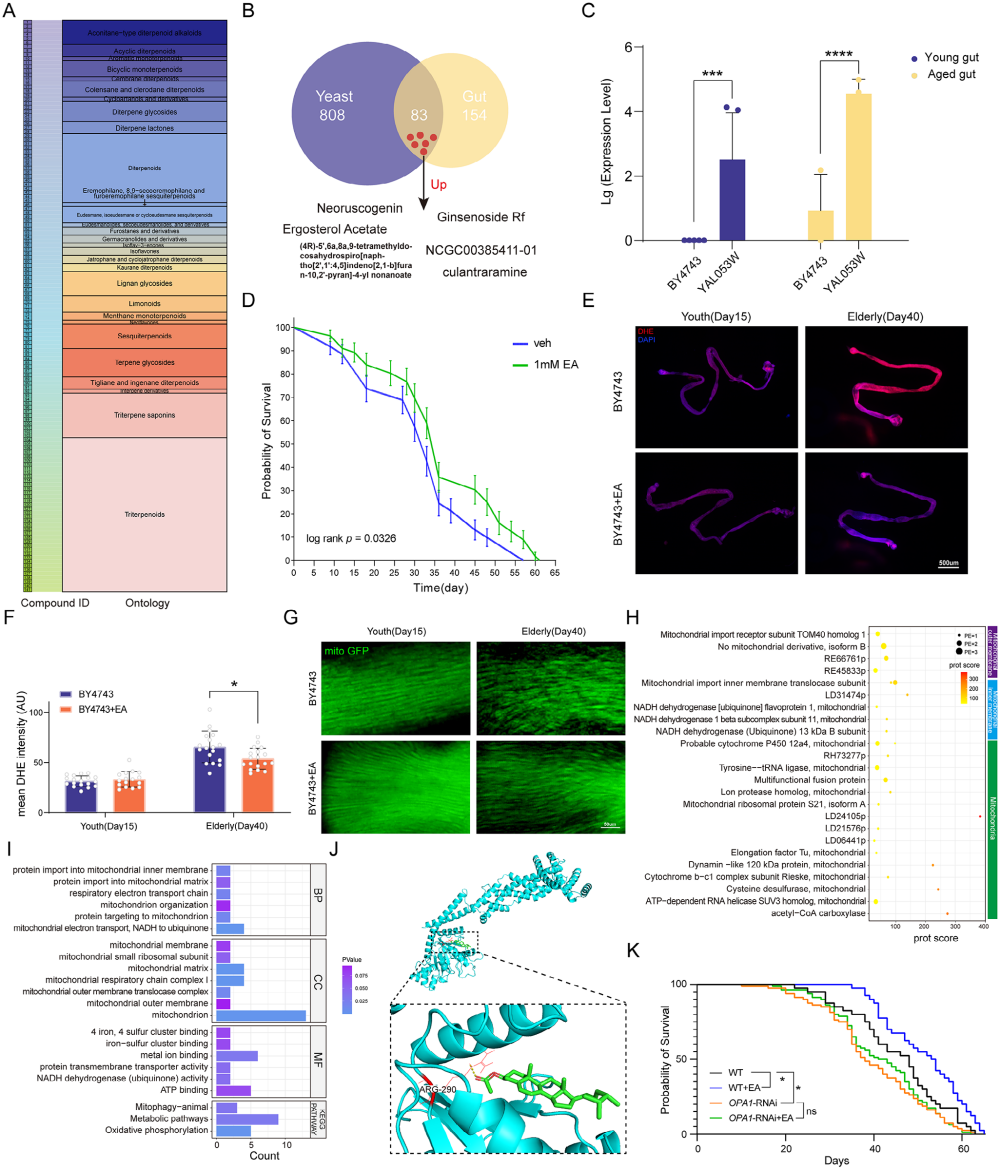
Roles of Terpenoid Compounds in Anti‐Aging Mechanisms. A) Chart showing 141 significantly upregulated terpenoid compounds and their associated Gene Ontology (GO) terms. Substance numbers correspond to Table S6 (Supporting Information). **B)** Venn diagram illustrating the number of terpenoid compounds present in both the *Drosophila* gut and *yeast* strains, along with the shared highly expressed compounds. **C)** Expression levels of ergosterol acetate in the gut of *Drosophila* fed the YAL053W mutant strain, including levels in both young and aged flies. The y‐axis represents the log10 (Lg) of ergosterol acetate expression. Significance was denoted by ^*^ *p* < 0.05, ^***^ *p* < 0.001, and ^****^ *p* < 0.0001 based on Student's *t*‐test. **D)** Survival curve of *fruit flies* fed with a 1 mm concentration of ergosterol acetate. Statistical significance based on Log‐rank test. **E)** ROS levels in the gut of young (day 15) and elderly (day 40) *Drosophila* fed with wild‐type control (*BY4743*) or EA. Higher ROS levels are indicated in red, and DAPI staining is shown in blue (4X magnification) (*n* = 6 × 3). **F)** Statistical analysis of fluorescence intensity and range in *Drosophila*. Average fluorescence intensity and range were quantified using ImageJ. *n* = 18; *p* < 0.05, as determined by Student's *t*‐test. **G)** Mitochondrial distribution in the flight muscles of *Drosophila* fed *BY4743* and EA at different ages. GFP‐labeled mitochondria are shown at 40X magnification (*n* = 6 × 3). **H)** Scoring of ergosterol acetate interactions with mitochondrial‐associated proteins in pull‐down experiments. PE indicates reliability, with lower scores (1–5) representing higher reliability. **I)** Bar chart depicting enrichment results of mitochondrial‐associated proteins. **J)** Virtual docking results of ergosterol acetate with *OPA1* protein. **K)** Survival curves of wild‐type (WT) fruit flies, WT fruit flies fed ergosterol acetate, *OPA1*‐silenced fruit flies, and *OPA1*‐silenced fruit flies fed EA. Statistical significance based on Log‐rank test. *n* = 80.

Terpenoids, renowned for their anti‐aging and anti‐inflammatory properties, are typically derived from rare and costly plants or fungi. In fungi alone, over 200 terpenoids have been identified.^[^ ^38^ ^]^ Compared to the control *yeast BY4743*, 141 terpenoids were significantly upregulated in mutant *S. cerevisiae* cultured in vitro (LogFC > 1, *p* < 0.05) (**Figure** **8**A; Table S6, Supporting Information). Terpenoids are key metabolites in *yeast*, with metabolism occurring both in vivo and in vitro. Moreover, *yeast*‐derived metabolites in the gut of *D. melanogaster* can undergo further modification, potentially generating novel compounds. Among the 808 terpenoids, 83 were identified in gut metabolism, with 6 displaying significantly higher expression in the gut of flies, both young and aged, fed with anti‐aging mutant *yeast* (Figure 8B). Particularly, ergosterol acetate and ergosterol showed a notably strong negative correlation (Figure S3A, Supporting Information). This suggests that the presence of ergosterol in acetic acid form may result from acid‐modifying mechanisms in the mutant *yeast*.

Targeted mass spectrometry revealed a significant increase in ergosterol acetate levels in flies fed with *YAL053W* mutant *yeast* compared to those fed with *BY4743* (Figure 8C). Notably, *Drosophila* fed with ergosterol acetate showed a 7.02% increase in lifespan (*p* < 0.05) compared to those fed with the wild‐type strain *BY4743* (Figure 8D). Intestinal ROS levels were significantly reduced in aged flies following EA treatment (Figure 8E,F). Additionally, EA‐fed flies exhibited more stable mitochondrial morphology (Figure 8G), further supporting the role of EA in mitigating age‐associated cellular deterioration.

To explore the mechanism underlying ergosterol acetate's lifespan‐extending effects, pull‐down experiments were performed using biotin‐labeled *fruit fly* proteins and ergosterol acetate standards (Figure S4A, Supporting Information). A total of 405 proteins were identified, primarily enriched in biological processes such as protein phosphorylation and mitochondrial electron transport. These proteins were predominantly localized to the mitochondria and exhibited molecular functions related to ATP binding (Figure S4B, Supporting Information). Of these, 24 proteins, localized to the inner and outer mitochondrial membranes, or associated with mitochondrial function, were identified as potential ergosterol acetate binding partners (Figure 8H). Enrichment analysis indicated their involvement in protein translocation, transmembrane transport, mitochondrial metabolism, and oxidative phosphorylation (Figure 8I).

Molecular docking and molecular dynamics simulations were conducted for the top five proteins interacting with ergosterol acetate, with both methods identifying *OPA1* as having the lowest binding free energy (Figure S5A–D, Supporting Information). The binding sites included the oxygen atom of ergosterol acetate and the amino acid ARG at position 290 of *OPA1* (Figure 8J). Recent studies have underscored the pivotal role of *OPA1* in maintaining mitochondrial protein stability, regulating cristae morphology, and stabilizing respiratory chain complexes.^[^ ^39^ ^]^ To further explore the role of *OPA1*, we silenced *OPA1* in fruit flies (Figure S5E, Supporting Information) and fed them ergosterol acetate. The results showed a significant reduction in lifespan in the *OPA1*‐silenced flies, with no lifespan extension observed after EA treatment (Figure 8K). Therefore, it is hypothesized that ergosterol acetate enhances mitochondrial stability by interacting with *OPA1*, a key regulator of cristae integrity, thereby decelerating the aging process.

## 3 Discussion

### 3.1 Genomic Engineering of Gut Microbiota: Manipulating Gut Microbial Genetic Variation to Prolong Host Longevity

Numerous studies have explored the impact of species and strain diversity on host longevity through microbiome composition; however, far less attention has been given to genetic variation within these microorganisms.^[^ ^40^, ^41^ ^]^ Recent research has shown that genetic variation within the gut microbiome is a critical factor influencing host longevity, playing a significant role in natural selection and biological diversity.^[^ ^42^ ^]^ The adaptability of the gut microbiota suggests that genetic variants offering benefits to the host are more likely to be propagated and retained. Lita Proctor et al. also emphasized that understanding the evolutionary dynamics between hosts and gut *S. cerevisiae* will be a key focus for future studies on intestinal flora.^[^ ^17^ ^]^

This study aims to thoroughly examine the influence of genetic variation in gut microbes on host longevity and activity, ultimately aiming to develop innovative microbial interventions to promote well‐being and extend lifespan. However, the complexity of gut microbiota and challenges in standardizing research protocols hinder efforts to study microbiome‐host interactions. Guo et al. proposed that simplifying the research by focusing on a single intestinal bacterium and conducting multi‐gene knockouts could offer novel insights into how gut microbial metabolites affect the host.^[^ ^43^ ^]^ To systematically evaluate the role of genetic variation in gut microbes on host longevity, motor performance, and reproduction, this study fed axenic *D. melanogaster* nearly 2000 individual homozygous diploid strains of single‐gene knockout *S. cerevisiae* to assess host lifespan. Our research identified 14 novel genes within *S. cerevisiae*, whose mutations significantly extended the lifespan of *fruit flies*. Notably, these mutant *yeast*s not only prolonged the longevity of the flies but also maintained their motor function. Notably, 13 of these mutants further delayed the age‐related decline in motor function. These discoveries offer valuable microbial resources for advancing anti‐aging research. The investigation was extended to assess the impact of different feeding windows on the longevity‐promoting effects of the *YAL053W* mutant strain. Flies exposed to the mutant yeast solely during the larval stage, with no subsequent supplementation after eclosion, did not exhibit a significant lifespan extension. However, continuous supply of the mutant strain from day 1 to day 7 of adulthood resulted in a notable lifespan extension. The longevity effect was particularly pronounced in flies fed the mutant strain throughout their entire lifespan (Figure S3B–D, Supporting Information). These findings indicate that early adult‐stage feeding can induce a significant pro‐longevity effect, but continuous exposure is necessary for maximal benefit, underscoring the critical role of timing and duration in microbial interventions modulating host aging.

### 3.2 The Gut Plays a Pivotal Role in the Regulation of Host Aging and Exhibits Sex‐Specific Effects

Research has revealed that rapamycin administration extends the lifespan of female *Drosophila* but has minimal effects on males. In contrast, genetic manipulation of the transformer gene in *Drosophila* specifically alters the sex identity of intestinal cells without influencing overall sexual identity. Notably, rapamycin‐treated male flies experience significant lifespan extension while maintaining their reproductive capacity.^[^ ^44^ ^]^ A related study in nematodes revealed that a mutation in the insulin signaling gene daf‐2 across all cells nearly doubled lifespan, though fertility was reduced by ≈30%. Conversely, when daf‐2 mutations were restricted to nerve tissue, no decline in fertility occurred, nor was there any increase in lifespan. Interestingly, nematodes with mutations only in intestinal tissue displayed a twofold lifespan extension without any compromise in reproductive ability.^[^ ^45^ ^]^

In our investigation, anti‐aging mutant *S. cerevisiae* demonstrated similar gender‐specific effects on the lifespan of *Drosophila*. These mutant *yeast* strains significantly prolonged lifespan while preserving reproductive function. In particular, the knockout of the *YAL044C* gene in *yeast* notably enhanced reproductive capacity at various developmental stages and effectively delayed reproductive aging. These findings highlight the gut as a key target for anti‐aging interventions and suggest promising avenues for future research into probiotics or drugs aimed at enhancing gut health to combat aging.

### 3.3 Changes in Metabolism and Proteome Balance of *D. melanogaster* Following Feeding on Anti‐Aging Mutant *Yeast*

The body's metabolism undergoes substantial restructuring with age, and the gut, as a key organ for nutrient metabolism and the primary hub for microbial‐host interactions, plays a central role in this process. Using quantitative mass spectrometry, significant metabolic remodeling was observed in the intestinal metabolism of young flies after consumption of anti‐aging mutant *yeast*. These metabolic alterations were linked to pathways such as primary bile acid biosynthesis, galactose metabolism, riboflavin metabolism, butanoate metabolism, and glycerolipid metabolism. Functional classification of these metabolites unveiled distinct regulatory mechanisms underlying biological traits like lifespan, locomotion, and reproduction, underscoring the complexity of anti‐aging regulation. The results suggest that modulating intestinal metabolic status through mutant *S. cerevisiae* could be a key strategy for improving host health; however, extending healthy lifespan by targeting a single metabolic pathway remains a challenge. Interestingly, the study found that mutant *yeast* improved the overall gut metabolic status in aged flies, leading to rejuvenation compared to control flies.

Beyond its impact on the intestinal metabolic state, mutant *yeast* also significantly influenced protein expression in tissues outside the gut. Notably, the upregulation of proteins in long‐lived flies far exceeded the downregulation. Moreover, over 50% of the long‐lived *Drosophila* displayed a strong correlation between upregulated proteins and mitochondrial energy metabolism. Mutant *yeast* also played a critical role in modulating key metabolic pathways tied to energy metabolism, further highlighting its potential in regulating host vitality and longevity.

### 3.4 The Energy Metabolism Pathway May Serve As a Pivotal Metabolic Pathway in the Realm Of Healthy Aging Research

During natural selection, organisms often face the trade‐off of delayed harm as a cost for early benefits such as enhanced reproductive capacity and shortened lifespan. As a result, a negative correlation between lifespan and reproduction has been observed across various species, including humans. Energy allocation plays a pivotal role in balancing reproduction and longevity.^[^ ^46^ ^]^ For example, the trl‐1 gene optimizes nutrient allocation across generations, regulating this trade‐off.^[^ ^29^ ^]^ It is hypothesized that the anti‐aging mutant *yeast* identified in our study may possess the unique ability to delay aging while preserving reproductive capacity by intricately modulating host energy metabolism, thus achieving a harmonious balance between reproduction and longevity. Discovering mechanisms that extend lifespan without compromising reproductive ability, and even delay reproductive aging, is of great importance. Rapamycin, an anti‐aging drug, acts through the mTOR signaling pathway, regulating energy allocation to maintain somatic cell functionality while diminishing reproductive function, thereby extending lifespan.^[^ ^47^ ^]^

### 3.5 Revelation of Novel Bioactive Compounds With Anti‐Aging Properties

Terpenoids, known for their anti‐cancer and anti‐aging properties, play a critical role in drug development.^[^ ^48^ ^]^ Engineering *yeast* has emerged as a key strategy for terpenoid synthesis.^[^ ^49^, ^50^ ^]^ Through metabolomic analysis of 14 mutant *yeast* strains and the examination of intestinal metabolites in *D. melanogaster* fed with these mutants, terpenoids were identified as potential anti‐aging compounds. Notably, ergosterol acetate, a metabolite of ergosterol, significantly extended the lifespan of *Drosophila*, whereas ergosterol itself did not (Figure S3E, Supporting Information). It is hypothesized that acetic modification enhances ergosterol acetate's interaction with host proteins. Our findings show that ergosterol acetate exhibits a stronger affinity for mitochondrial‐associated proteins, particularly the mitochondrial membrane protein *OPA1*,^[^ ^39^ ^]^ as confirmed by molecular docking and kinetic simulations. Additionally, ergosterol acetate's greater lipid solubility, compared to ergosterol, facilitates its superior ability to traverse cell membranes. Despite being essential for mitochondrial membrane stability and viability in *yeast*,^[^ ^51^, ^52^ ^]^ ergosterol does not contribute to lifespan extension in *D. melanogaster*.

*Yeast* is rich in bioactive compounds with anti‐aging properties, making it a central focus in anti‐aging research.^[^ ^11^, ^53^ ^]^ The development of genetically modified strains with enhanced anti‐aging effects holds significant potential for advancing the field. In this study, 14 novel mutant strains with remarkable anti‐aging efficacy were identified through an extensive screening of ≈2000 *S. cerevisiae* gene knockout strains. Moreover, ergosterol acetate, one of the key active components linked to these anti‐aging benefits, was successfully characterized. As mitochondrial membrane stability declines with age, impairing mitochondrial energy metabolism,^[^ ^54^ ^]^ the mutant *S. cerevisiae* may produce significant amounts of ergosterol acetate in older hosts, stabilizing mitochondrial membranes and enhancing energy metabolism, thus contributing to anti‐aging effects.

In conclusion, the discovery of specific *yeast* mutations and their associated metabolites, along with insights into their mechanisms of action, could lead to the development of novel anti‐aging drugs or therapies. This study highlights the promising potential of targeting intestinal metabolism as a strategy for anti‐aging interventions. While the findings emphasize the role of microbial genetic variation in Drosophila aging, caution is required when translating these results to human applications. Drosophila provides a valuable model for initial mechanistic insights; however, interspecies differences in gut microbiota composition, host immunity, and metabolic regulation necessitate further validation in mammalian systems before clinical applications can be considered.

## 4 Experimental Section

### *D. Melanogaster, S. Cerevisiae* Strains and Culture

The *D. melanogaster* Oregon K strain (isogenic wild‐type) was obtained from the Core Facility of *Drosophila* Resource and Technology at CEMCS, CAS. The *S. cerevisiae* gene‐knockout collection (YKOC) was procured from Invitrogen in 2014. Axenic *Drosophila* was prepared using a method described in a previous study.^[^ ^55^ ^]^ The *Drosophila* culture medium was created by boiling a mixture of superfine corn flour, agar powder, glucose, sucrose, *yeast*, and deionized water for 30 min, followed by the addition of 4 mL of propionic acid. *S. cerevisiae* was grown in YPD medium (*Yeast* extract, Peptone, Dextrose) until reaching the stationary phase (OD_600_ = 1.8–2.0) after selecting a single colony. For longevity and feeding assays, 35 mL of stationary‐phase *S. cerevisiae* was centrifuged, resuspended in 200 µL of PBS, and added to tubes containing the regular culture medium for adult *D. melanogaster*. Additionally, solid YPD medium was prepared by adding 2% agar to liquid YPD medium. UAS‐mito‐GFP Drosophila (purchased from the Tsinghua Fly Center) and GSTD1‐GFP Drosophila (a generous gift from Professor Wei Liu at our institution) were maintained under the same conditions as the wild‐type flies.

*OPA1* gene silencing was achieved using the UAS‐RNAi‐*opa1*‐like line (THU0811) and the tub‐GAL4 driver line (TB00129), both obtained from the Tsinghua Drosophila Stock Center. The UAS‐RNAi‐*opa1*‐like males were crossed with tub‐GAL4 females to generate the progeny. The resulting offspring with normal phenotypes were selected for lifespan experiments.

### Colonization of the Strain‐Exposure to YPD Medium

Male flies of specific ages (3–5 individuals) were selected based on experimental needs. Flies were thoroughly rinsed with distilled water to remove contaminants, and the heads and bodies were separated to expose the gut to a YPD solid medium. Cultures were incubated at 30 °C for 24 h, and colony growth was documented using a camera.

### Colony‐Forming Unit (CFU) Assay

Wild‐type flies within 8 h of eclosion were sexed and transferred to vials containing different *S. cerevisiae* mutant strains. On days 15 and 40, six flies from each group were randomly selected for gut CFU analysis. Under sterile conditions, whole intestines were dissected in 1 mL sterile PBS, sectioned into four segments, and vortexed for 30 s. A 50 µL aliquot of the supernatant was plated onto YPD agar and incubated at 30 °C for 48 h before colony counting.

Yeast strains were pre‐cultured in 1 mL YPD at 30 °C, 200 rpm for 12–16 h to reach mid‐log phase, then inoculated into fresh YPD at 1% (v/v; e.g., 0.3 mL into 30 mL). Immediately after inoculation, 1 mL was taken for OD_600_ measurement and serial dilution in sterile saline for CFU plating on fly‐standard agar. Plates were incubated at 25 °C for 48 h before colony counting. Cultures were grown at 30 °C, 200 rpm, and sampled every 4 h–24 h, with OD_600_ measured and CFU determined.

### Longevity Assay

On Day 0 (the day of eclosion and gender selection), 80 adult male virgin flies were transferred into new tubes, each containing a different strain of *S. cerevisiae* (20 flies per tube, 4 tubes per group). To ensure consistent yeast feeding throughout the lifespan of the flies, food and yeast strains in the tubes were refreshed every 3–5 days. Fly mortality was recorded daily until all flies expired. Each *yeast* strain's lifespan experiment was replicated three times, and averages were used for statistical analysis and survival curve generation.

### Climbing Assay

For climbing assays, 80 male flies selected at youth and midlife stages were evenly distributed into four test tubes. After a 5 min acclimation period, the tubes were gently tapped to ensure all flies were at the bottom. The number of flies that climbed 6 cm within 10 s was recorded. Each tube underwent five trials, and the climbing index was calculated as the proportion of flies that successfully climbed 6 cm within the allotted time, relative to the total number of flies.

### Gut Barrier Assay

Young and aged *Drosophila*, after consuming the mutant strain, were transferred to new tubes containing a medium mixed with Brilliant Blue staining solution at a concentration of 2.5 g/100 mL. Following a 9 h incubation period, the percentage of *Drosophila* exhibiting “Smurf” coloration was assessed under a microscope.

### Reproductive Assay

Newly eclosed *Drosophila* (Day 0) were sexed on a flight board, and groups of 5 males and 5 females were placed in each vial. The flies were fed mutant *yeast* strains according to the same protocol used in the lifespan assay. Each day, flies were transferred to fresh vials at the same time, with the previous vial retained. After 10 days, the number of flies from the old vial was counted to assess total egg production for that day.

### Biochemical Composition and Energy Content Analyses of Yeast Strains‐Protein Quantification

Protein concentration of yeast samples was determined using the Enhanced BCA Protein Assay Kit (Cat. No. BL1054A, Biosharp). Yeast cultures were initiated from a single colony grown in 1 mL YPD medium at 30 °C, 200 rpm for 12–16 h, then transferred to 30 mL fresh YPD for an additional 18 h until OD_600_ reached 1.8–2.0. After centrifugation at 7000 rpm for 5 min, the cell pellet was resuspended and diluted 1:100 in PBS. Samples (20 µL) were added to a 96‐well plate, mixed with 200 µL of freshly prepared BCA working reagent (Reagent A:Reagent B = 50:1), and incubated at 37 °C for 30 min. Absorbance at 562 nm was measured using a microplate reader. A standard curve was generated using serial dilutions of bovine serum albumin (BSA) to calculate sample protein concentrations.

### Total Sugar Content Measurement

Total sugar content was measured using a colorimetric Total Sugar Assay Kit (Cat. No. ADS‐F‐TDX030, Adamas). Yeast cultures were prepared as described above, and 0.1 mL of the cell suspension (diluted 5–10 fold in PBS) was treated with 400 µL of Reagent I (a 1:1 mix of 36%–38% HCl and PBS), incubated at 90 °C for 30 min with intermittent shaking, and then cooled to room temperature. Subsequently, 400 µL of Reagent II was added, and the solution was adjusted to a final volume of 1 mL with distilled water. After centrifugation at 12 000 rpm for 10 min at 25 °C, the supernatant was collected. For detection, 100 µL of the supernatant was mixed with 100 µL of Reagent III, heated at 95 °C for 10 min, rapidly cooled, and then mixed with 1 mL of distilled water. Absorbance was measured at 500 nm. Total sugar concentration was calculated based on the standard curve Eq. (1):

(1) y=10.411x−0.0084

where. *x* = sugar content (mg),*y* = ΔA"

### Crude Fat Determination

Crude fat content was determined by Soxhlet extraction. Yeast pellets were collected as described and dried at 105 °C to a constant weight. Approximately 0.5–1 g of dried sample was placed into a filter paper thimble and extracted with anhydrous ether (or petroleum ether) for 6–10 h in a Soxhlet apparatus, with a reflux rate of 6–8 cycles per hour. After extraction, the solvent was evaporated, and the remaining lipid was dried at 100 °C for 1 h to a constant weight. Crude fat percentage was calculated as: Crude fat (%) = (mass after extraction − mass after drying) / initial sample mass × 100

### Calorimetric Energy Determination

The caloric content of yeast biomass was measured using an oxygen bomb calorimeter. Cultured yeast cells were harvested, dried, and weighed (0.2 g), then sealed in a combustion capsule. The bomb was charged with oxygen to 2.5–3.0 MPa and immersed in a calorimeter containing 2000–3000 mL of distilled water. After baseline temperature stabilization, combustion was initiated. Temperature was recorded before, during, and after combustion until a stable maximum was reached. Energy release (caloric value) was calculated based on the change in water temperature and the heat capacity of the calorimeter. The results were expressed as energy content per gram of dry biomass (kcal/g or J/g).

### Mitochondrial Fluorescence Detection in *Drosophila*

For flight muscle mitochondrial fluorescence detection, several GFP‐labeled *Drosophila* were collected and placed in pre‐cooled PBS. Under a stereomicroscope, heads and tails were removed, and the thorax was dissected. The dorsal muscles were carefully extracted, placed on a slide, washed 2–3 times with PBS, and sealed with glycerol and nail polish. Fluorescence microscopy was used to examine the samples. For fat body mitochondrial fluorescence detection, GFP‐labeled *Drosophila* were collected and placed in pre‐cooled PBS. Under a stereomicroscope, the heads and thorax were removed, and the tail was gently opened to reveal the fat body. A single layer of fat body tissue was isolated, placed on a slide, washed 2–3 times with PBS, and stained with DAPI for 10 minutes. The tissue was then washed three times with PBS for 5 min each. After glycerol was added, the slide was sealed with nail polish and examined under a fluorescence microscope. Fluorescence images were analyzed and quantified using ImageJ software.

### ROS Detection in *Drosophila* Intestinal Tissue

*Drosophila* samples were dissected to assess intestinal ROS levels as follows: flies were immobilized in PBS, with heads and tails removed, and thoraxes positioned to expose the distal intestine. After careful dissection to isolate the intestine and remove associated tissues, the samples were mounted on glass slides. ROS levels were assessed using the dihydroethidium (DHE) staining assay. Samples were fixed in 4% paraformaldehyde for 15 min at room temperature and washed three times with PBS. Tissues were then incubated with 60 µm DHE in the dark for 10 min, followed by another PBS wash. Nuclear staining was performed using 5 µg mL^−1^ DAPI for 10 min, after which samples were washed and mounted in glycerol. Imaging was performed within 1 h using a fluorescence microscope, with consistent exposure settings across all groups. DHE fluorescence intensity in the intestinal epithelium was quantified using ImageJ software, and the mean fluorescence intensity was used as a measure of ROS levels.

For the GSTD1‐GFP *Drosophila*, the flies were anesthetized and placed on a fly pad for rapid imaging under a fluorescence microscope, ensuring identical parameter settings for experimental and control groups within each batch to reduce variability in fluorescence intensity. ImageJ software was used to calculate the average fluorescence intensity and fluorescence area.

### Metabolomics Sequencing in *Drosophila* and *Yeast*

Young (Day 15) and old (Day 40) male *fruit flies*, fed with mutated *S. cerevisiae* strains, were selected for the study. The flies were thoroughly rinsed with distilled water to remove any impurities and dissected in pre‐chilled PBS to extract the entire intestines along with their contents (excluding the crop), which were immediately frozen for storage. Five tubes were prepared for the young group, and three tubes for the old group, due to the fragility of the intestines, with each tube containing 10–20 intestines. The remaining fly bodies after gut dissection were collected as extraintestinal tissue samples for subsequent proteomic analysis. Additionally, 5 mL of yeast cultures in the stationary phase (OD600 = 1.8–2.0) were collected and submitted for metabolomic profiling, with six replicates per sequencing group.

The samples were sent for untargeted metabolomics analysis, following the methodologies of Lee et al.^[^ ^56^ ^]^ and Roberts et al.,^[^ ^57^, ^58^ ^]^ using LC‐MS in positive ion mode. The raw data were processed and identified using MSDIAL, with annotations based on public databases. After quality control and calibration, a peak area expression matrix was generated, excluding unidentified metabolites. For duplicated metabolites, the one with the highest average intensity was selected for further analysis. The output results were imported into R for subsequent calculations.

### Data Processing and Statistical Analysis for Metabolomics

The raw metabolite expression levels were processed using the MetaboAnalystR R package.^[^ ^59^, ^60^ ^]^ PCA was performed to identify differences between the control group (BY4743) and *fruit fly* intestines corresponding to the 14 mutant strains in the experimental group. For clarity, the intestines of flies fed with mutated *S. cerevisiae* strains were labeled according to the mutant genes. Data normalization was conducted using the MetaboAnalystR package with Quantile normalization to assess the normal distribution. Log transformation was applied to correct sample variation, and Mean Centering was performed to allow for a comprehensive examination of individual features. Significance was defined by a *p* < 0.05, and differential expression was determined by an absolute |Log2(Fold Change)| > 1.

To further explore metabolite‐phenotype associations, the Weighted Gene Co‐expression Network Analysis (WGCNA) package^[^ ^61^, ^62^ ^]^ was employed. Enrichment analysis was carried out via the MetaboAnalyst platform (https://www.metaboanalyst.ca/), with visualizations generated using the R language. Scatter density plots were created using the ggplot2 and ggdensity R packages, while correlation plots were constructed with the corrplot package. Chord diagrams were produced using the GOplot package, and differential metabolites and associated pathways were visualized using FlyScape. In these visualizations, metabolites were represented by hexagons, with red hexagons indicating metabolites of interest, while gray diamonds represented related reactions.

### Proteomic Sequencing of *Drosophila* Non‐Intestinal Tissues

Samples from non‐intestinal tissues of young male *Drosophila*, previously utilized in metabolomic sequencing, were analyzed. The protein spectra obtained through mass spectrometry were processed for differential expression using R, and bubble plots were used to visualize the results (Five replicates per sequencing group).

### Ergosterol Acetate Biotin Labeling Pull‐Down Assay and Molecular Docking

In the biotin‐labeled pull‐down assay and molecular docking of ergosterol acetate, adult wild‐type *Drosophila* tissues and ergosterol acetate standards underwent biotinylation, followed by desulfurization of ergosterol acetate and total protein extraction. Proteins were conjugated with ergosterol acetate, incubated, and affinity‐purified using streptavidin‐conjugated magnetic beads to eliminate nonspecific interactions. The washed protein complexes were analyzed to identify specific interactions with the small molecule fragment. Docking studies were conducted using AutoDock, with receptor proteins sourced from the PDB database and the molecular structure of ergosterol acetate obtained from PubChem. The process included dehydration, hydrogenation, and setting active binding pockets in AutoDock, followed by docking via Vina. The docking results with the lowest affinity were visualized in PyMOL, highlighting the binding sites.

### Molecular Dynamics Simulations

Molecular dynamics simulations were employed to confirm the binding stability between ergosterol acetate and its potential target proteins, which showed strong binding affinities during docking. The simulations were conducted using GROMACS 2022.6 version.^[^ ^63^ ^]^ Protein topologies were generated by the pdb2gmx module of GROMACS, utilizing the amber14sb force field and TIP3P water model. Ligand topologies were prepared using the ACPYPE online platform (https://www.bio2byte.be/acpype/submit). The system was solvated in a dodecahedron box with a 1.0 nm distance from the ligand‐protein complex, and neutralized by adding NaCl. Energy minimization was performed using the steepest descent integrator for 5,000 steps. Equilibration was carried out for 100 ps under isothermal‐isochoric (NVT) and isothermal‐isobaric (NPT) ensembles. Finally, a 100 ns molecular dynamics simulation was run with a 2 fs time step. Simulation trajectories were analyzed using GROMACS tools, and binding free energies for the protein‐ligand complexes were calculated using the gmx_MMPBSA tool.^[^ ^64^ ^]^

### qPCR Analysis

Individual WT and *opa1* RNAi flies were collected in PBS and homogenized. Total RNA was extracted using standard TRIzol‐based procedures, and 1 µg RNA was reverse‐transcribed into cDNA using a commercial reverse transcription kit according to the manufacturer's instructions. qPCR was carried out in 20 µL reactions containing 2 µL cDNA, 10 µL SYBR Green premix, 0.8 µL primer mix (10µm forward and reverse primers), and 7.2 µL nuclease‐free water. Amplification was performed on a Roche LightCycler 480II system. Relative *opa1* expression levels were determined using the ΔCt method, normalized to *Rpl32* as the internal reference gene.

Primers:

*opa1*‐F: 5′‐CTCTGAGCACCAAGCTAT‐3′

*opa1*‐R: 5′‐GGCGCAACTTGATGTCTA‐3′

*Rpl32*‐F: 5′‐GACGCTTCAAGGGACAGTATC‐3′

*Rpl32*‐R: 5′‐AAACGCGGTTCTGCATGAG‐3′

### Statistical Analysis

Statistical analyses were performed using Student's t‐test in SPSS 19.0, with data presented as mean ± standard deviation. Between‐group comparisons were calculated as mean ± standard error of the mean (SEM). If normality assumptions were not met, non‐parametric Wilcoxon tests were applied. *Drosophila* survival curves were analyzed using the Kaplan–Meier method, with log‐rank tests used for comparison. Bar graphs and scatter plots were generated using GraphPad Prism 9.

## Conflict of Interest

The authors declare no conflict of interest.

## Author Contributions

L.W., Z.Y., Y.P., X.W. contributed equally to this work. Z.B., C.H., and Y.L. designed the experiments, conducted data analysis, and drafted the manuscript. L.W., Z.Y., Y.P., X.W., and P.Z. performed the majority of the experiments. X.Z., T.Z., Y.Z., X.D., D.Z., J.Z., and Z.B. discussed the data and its interpretation. L.W. and Y.Z. carried out metabolomics and proteomic detection as well as data analysis. W.L. provided technical support throughout the study. The final manuscript was reviewed and approved by all authors.

## Supporting information



Supporting Information

Supporting Information

Supporting Information

Supporting Information

Supporting Information

Supporting Information

Supporting Information

## Data Availability

The data that support the findings of this study are available in the supplementary material of this article.
