# Supplementary material for: Gut Microbial Genetic Variation Prolongs Host Healthy Longevity and Remodels Metabolome and Proteome in Drosophila Melanogaster
Source: Adv Sci (Weinh). 2025 Sep 26;12(47):e05469. doi: 10.1002/advs.202505469 (PMC12713025; doi:10.1002/advs.202505469)
Supplement: Supplementary file 1 — Supporting Information [file ADVS-12-e05469-s003.docx]

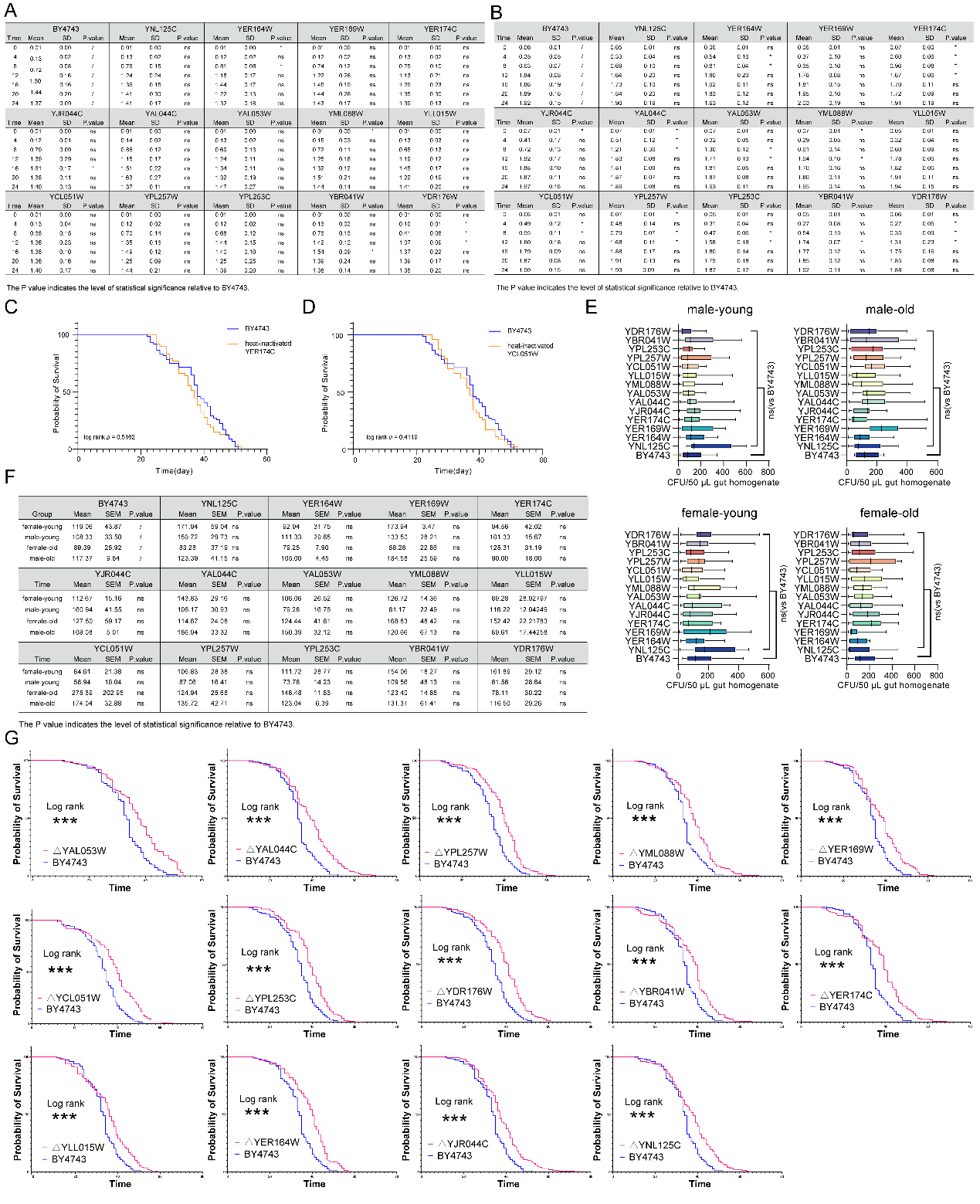


**Figure. S1: A:** Table presenting colony-forming unit (CFU) counts for 14 mutant yeast strains and the wild-type control strain *BY4743* at different time points. Statistical comparisons were performed using Student’s t-test; * denotes *P* < 0.05. **B:** Table displaying optical density at 600 nm (OD_600_) for 14 mutant yeast strains and the wild-type control strain *BY4743* at different time points. Statistical comparisons were performed using Student’s t-test; * denotes *P* < 0.05. **C:** Survival curve of *Drosophila* fed with heat-inactivated *YER174C* strain. Statistical comparisons were performed using the log-rank test. **D:** Survival curve of *Drosophila* fed with heat-inactivated *YCL051W* strain. Statistical comparisons were performed using the log-rank test. **E:** CFU counts from gut homogenates of male and female *Drosophila* at young and elderly stages. Statistical comparisons were performed using Student’s t-test; ns indicates no significant difference (*P* ≥ 0.05). **F:** Table summarizing CFU counts from gut homogenates of male and female *Drosophila* at young and elderly stages. **G:** Survival curves of *Drosophila* fed with each of the 14 mutant yeast strains compared to those fed with the wild-type control strain *BY4743*. Statistical comparisons were performed using the log-rank test; ****P* < 0.001.


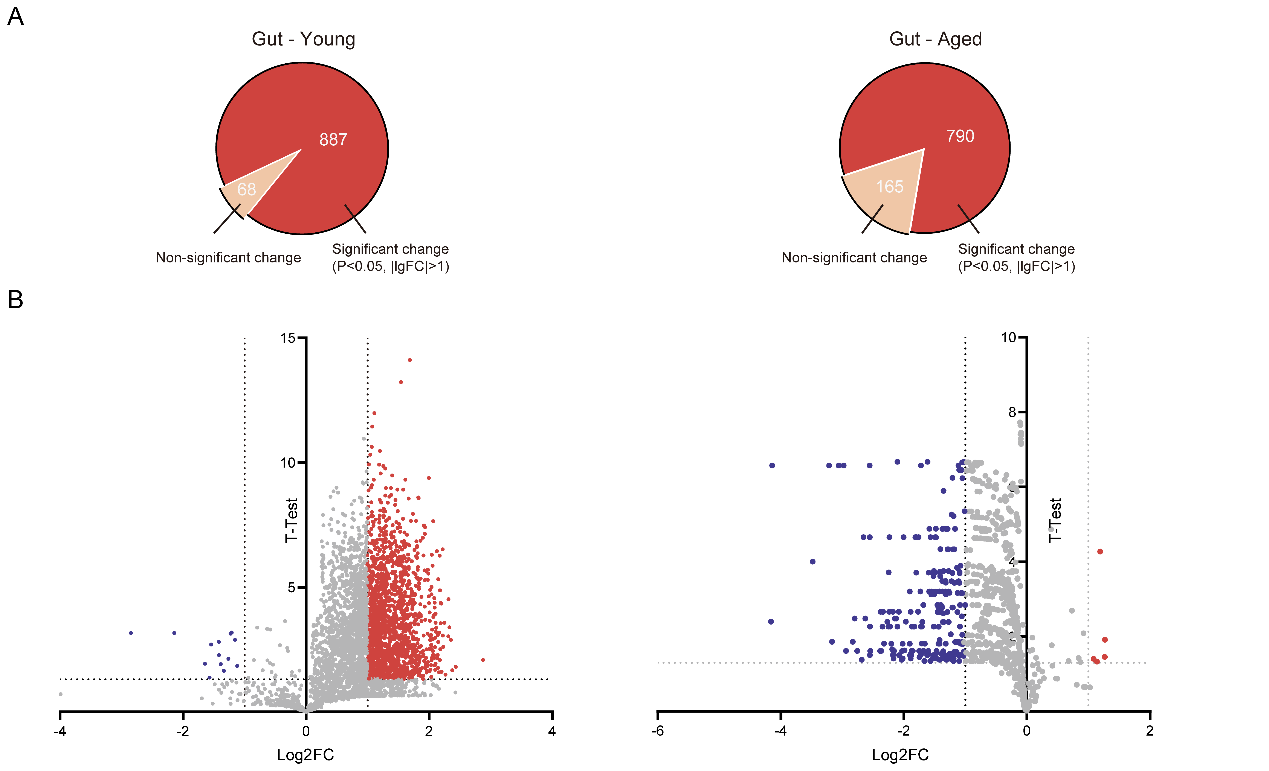


**Figure. S2:** **A:** Pie chart showing the abundance changes in metabolites in *Drosophila* fed with 14 anti-aging mutant *yeast* strains compared to the control strain. A total of 887 metabolites exhibited significantly increased abundance in the young stage, while 790 metabolites showed significantly decreased abundance in the aged stage (*P* < 0.05, |LogFC| > 1). **B: Metabolite changes during the older stages of *Drosophila* longevity.** Volcano plot illustrating the extent of up-regulated metabolites in aging *Drosophila*, comparing changes in *Drosophila* longevity during old age relative to control young flies. Volcano plot illustrating the extent of down-regulated metabolites in aging *Drosophila*, comparing changes in *Drosophila* longevity during old age relative to control young flies.


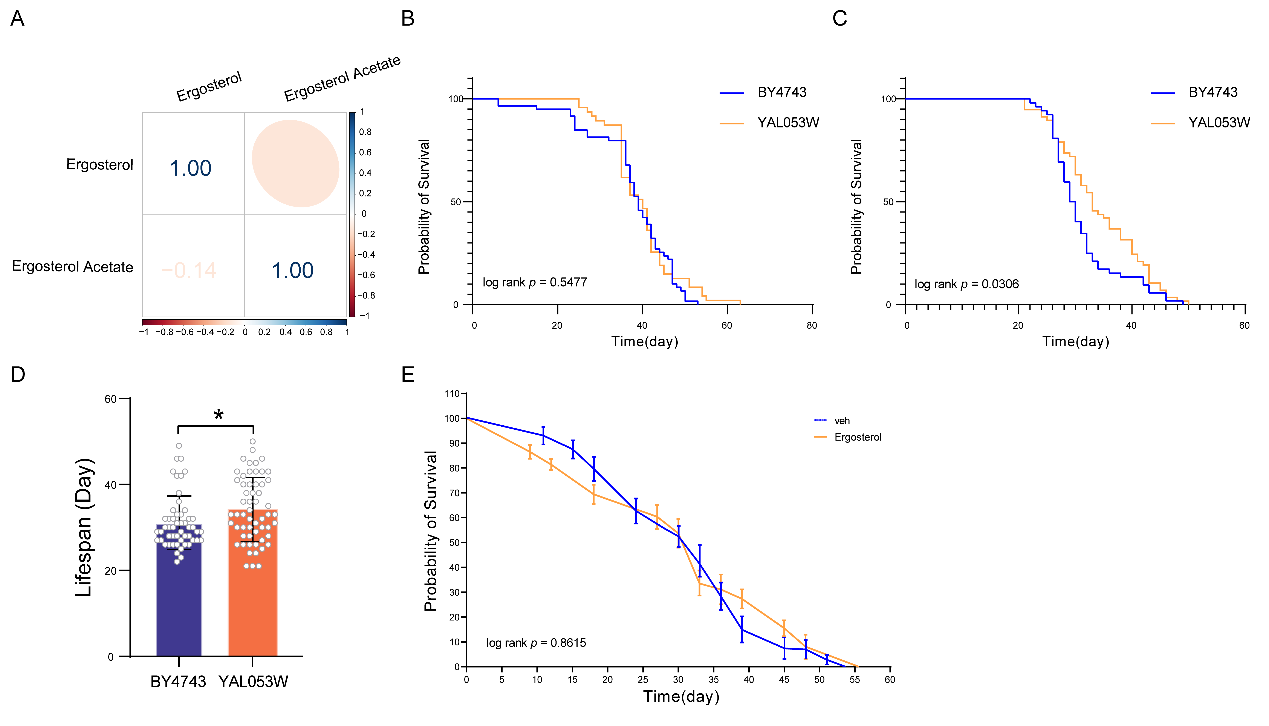


**Figure. S3: A:** Correlation analysis between ergosterol and ergosterol acetate. **B:** Survival curves of *Drosophila* larvae fed with the *YAL053W* strain or the wild-type control strain *BY4743*. **C:** Survival curves of adult *Drosophila* fed with the *YAL053W* strain or the wild-type control strain *BY4743* only during the first week after eclosion. **D:** Lifespan statistics of adult *Drosophila* fed with the *YAL053W* strain or the wild-type control strain *BY4743* only during the first week after eclosion. Data are presented as mean ± SEM (n = 80). Statistical comparisons were performed using Student’s t-test; *P* < 0.05 is indicated by *. **E:** Survival curve of *Drosophila* fed with ergosterol. n=80. Error bars represent the standard error of the mean (SEM) for each time point. Statistical significance was assessed using the Log-rank test.


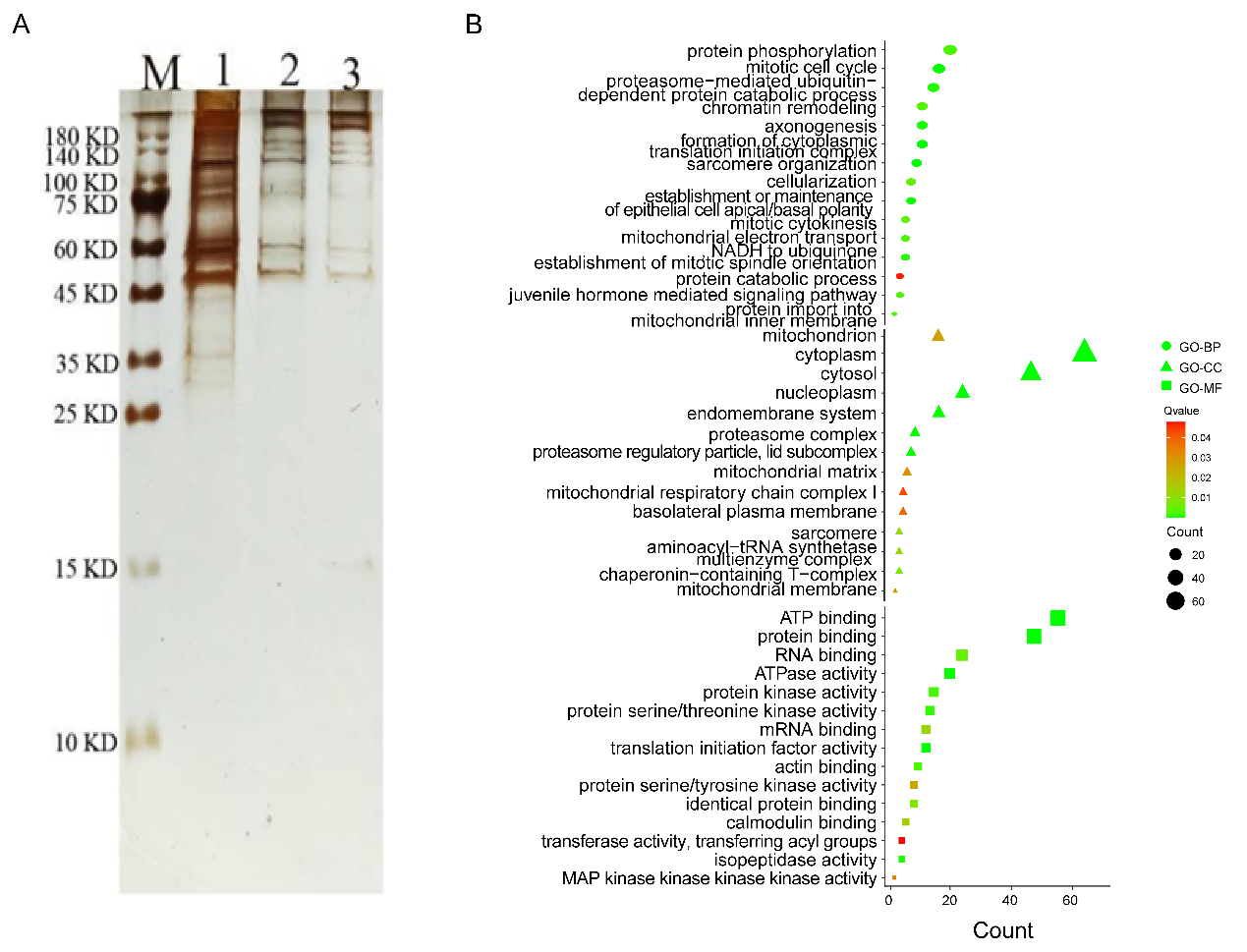


**Figure. S4: A:** SDS-PAGE analysis of biotinylated ergosterol acetate. Lane M: Protein marker, Lane 1: Ethyl α-D-glucopyranoside acetate + Bioeast Mag-SA + Total Protein, Lane 2: Biotin + Bioeast Mag-SA + Total Protein, Lane 3: Bioeast Mag-SA + Total Protein. **B:** GO enrichment bubble plot of proteins matched with ergosterol acetate.


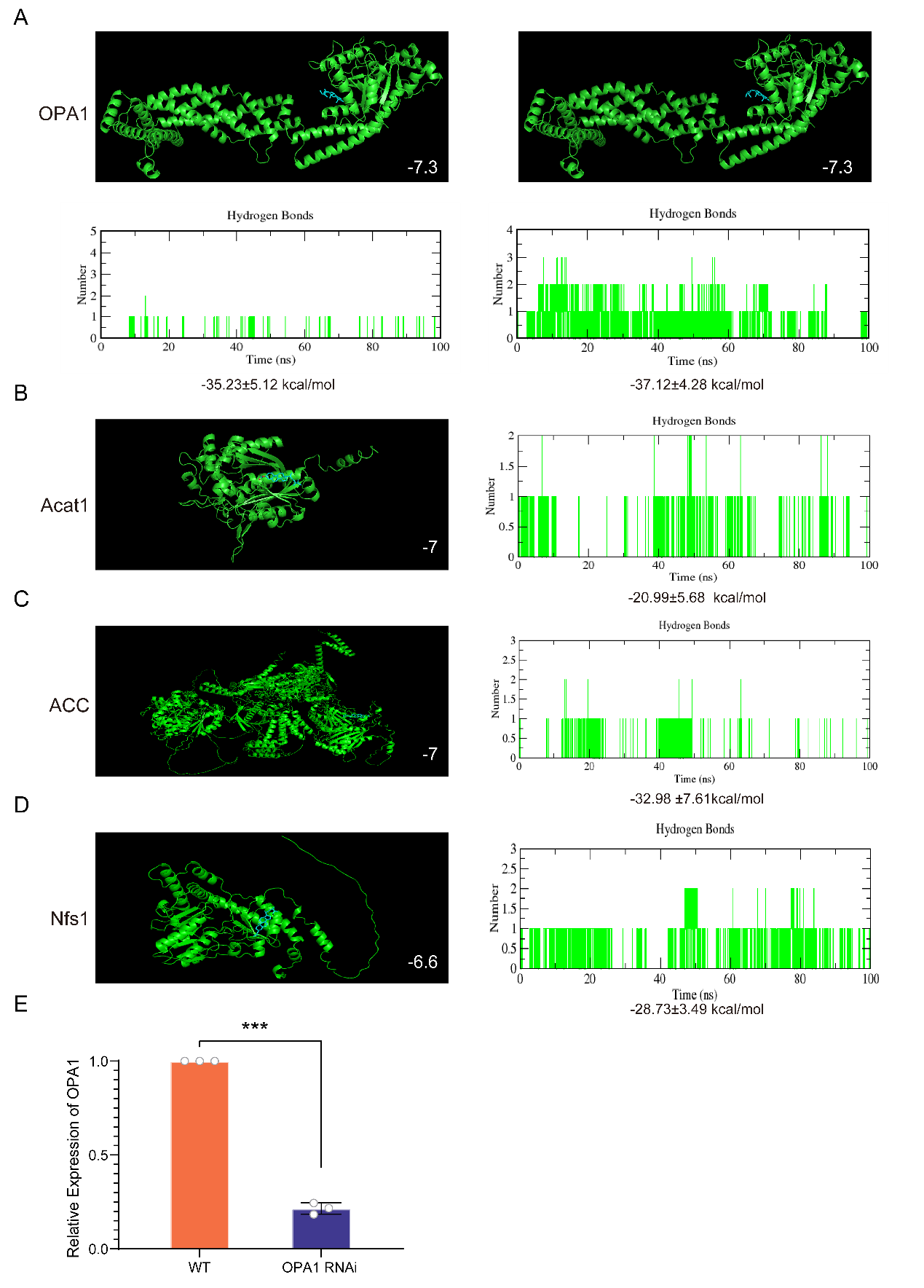


**Figure. S5: A, B, C, D:** Molecular docking and molecular dynamics simulation results of proteins *OPA1*, *Acat1*, *ACC*, and *Nfs1* with ergosta-7,22-dien-3β-ol. The docking scores (binding affinity, kcal/mol) are indicated in the bottom right of each docking schematic. The binding free energy values from molecular dynamics simulations (kcal/mol) are displayed below the hydrogen bond count plots. **E:** qPCR analysis of *OPA1* expression in WT and RNAi flies. Mean ± SEM; ****P* < 0.001, t-test.
